# ClpC1-dependent iron homeostasis underlies mycobacterial defense against oxygen-driven Fenton reaction during reactivation

**DOI:** 10.1128/spectrum.00496-26

**Published:** 2026-06-18

**Authors:** Mingzhe Chi, Yuqing Zheng, Wenkai Zheng, Youran Chen, Bohong Zhou, Jiayu Chen, Jiacheng Bai, Yuchang Di, Xuelian Zhang

**Affiliations:** 1 State Key Laboratory of Genetics and Development of Complex Phenotypes, School of Life Sciences, Institute of Infection and Health, Fudan University 12478, Shanghai, China; 2 Infectious Disease Department, Shenzhen Children’s Hospital 629292 , Shenzhen, People’s Republic of China; 3 Department of Infectious Diseases, Shanghai Key Laboratory of Infectious Diseases and Biosafety Emergency Response, National Medical Center for Infectious Diseases, Huashan Hospital, Fudan University 12478, Shanghai, China; 4 Shanghai Sci-Tech Inno Center for Infection & Immunity, Shanghai, China; 5 MOE Engineering Research Center of Gene Technology, Shanghai Engineering Research Center of Industrial Microorganism, School of Life Sciences, Fudan University 12478, Shanghai, China; University of Hawaii at Manoa, Honolulu, Hawaii, USA

**Keywords:** *Mycobacterium*, Clp protease system, iron homeostasis, oxygen reactivation, drug targets

## Abstract

The caseinolytic protease (Clp) system, which is highly conserved and essential for protein homeostasis in mycobacteria, comprises the ClpP1P2 proteolytic core and the essential ATPases ClpC1 and ClpX. While ClpX has a defined role in DNA replication, and some differential substrates have been identified, the physiological functions distinguishing ClpC1 from ClpX remain incompletely understood. Here, using Clustered Regularly Interspaced Short Palindromic Repeats-mediated knockdown in *Mycolicibacterium smegmatis* and the attenuated *Mycobacterium tuberculosis* H37Ra strain, we showed that ClpX impairment caused complete growth arrest, cell filamentation, and dramatically increased intracellular reactive oxygen species (ROS). In contrast, *clpC1* knockdown resulted in only moderate growth defects but induced profound hypersensitivity to high oxygen and catalase. Mechanistically, ClpC1 maintained iron homeostasis; its loss elevated free Fe²^+^ levels, driving Fenton chemistry, catastrophic ROS bursts, and redox collapse upon oxygen exposure. This iron-dependent survival defect was confirmed in a murine model. We thus establish ClpC1 as a central regulator of iron metabolism and oxidative defense, implicating its roles in bacterial reactivation from dormancy and persistence under fluctuating oxygen conditions. Furthermore, knockdown of either *clpC1* or *clpX* increased mycobacterial susceptibility to multiple antibiotics, highlighting the Clp system as an attractive anti-tuberculosis drug target. Together, our findings reveal overlapping yet non-redundant roles for ClpC1 and ClpX, delineating their distinct regulatory networks. These results advance our understanding of the Clp system. Importantly, although these conclusions are derived from studies in surrogate mycobacterial species, they raise the possibility that similar mechanisms may underlie adaptation, dormancy, and resuscitation in pathogenic *Mycobacterium tuberculosis*.

**IMPORTANCE:**

Tuberculosis remains a major global health burden. The ClpC1/ClpX-ClpP1P2 proteolytic complex has emerged as a highly attractive drug target. This study investigates the role of ClpX in maintaining fundamental bacterial biological processes and suggests a specific function in regulating iron homeostasis and oxidative defense. This proposed mechanism may contribute to the bacterium’s ability to survive environmental shifts, such as resuscitation from latency. By demonstrating an involvement of caseinolytic proteases (Clps) in iron metabolism, this research provides additional insights into mycobacterial adaptive strategies within the host. Importantly, the results highlight a putative vulnerability during latent infection and reactivation: mycobacteria appear to depend on ClpX and ClpC1 for dormancy regulation, while ClpC1 is likely important for mitigating reoxygenation-induced oxidative stress. This discovery may inform future therapeutic approaches specifically targeting the reactivation phase of latent tuberculosis.

## INTRODUCTION

Tuberculosis (TB) is a serious infectious disease caused by *Mycobacterium tuberculosis* (Mtb). Although TB is generally curable, the emergence of multiple drug-resistant (MDR) and extensively drug-resistant (XDR) strains has made the disease increasingly difficult to eliminate (1). Furthermore, Mtb’s ability to persist within the host for extended periods results in latent TB infection, which can reactivate when the immune system is compromised (2, 3). Reactivation of latent Mtb infection poses a significant barrier to global TB eradication efforts, as it accounts for a substantial proportion of newly emerging active TB cases. Reactivated TB not only perpetuates disease transmission but is also frequently associated with increased rates of drug resistance and unfavorable clinical outcomes (4). Consequently, there is an urgent need to develop new drugs or enhance the efficacy of current treatments, particularly against these drug-resistant strains, latent infection, and reactivating TB. The caseinolytic proteases (Clps) of Mtb have emerged as a highly novel drug target in recent years (5).

Clps are unique oligomeric serine protease complexes composed of a barrel-shaped peptidase (ClpP), flanked on one or both ends by a hexameric regulatory ATPase, such as ClpX, ClpA, or ClpC1, which together serve as key regulators of protein turnover and proteome homeostasis (6). Unlike most bacteria, which encode a single ClpP ortholog, Mtb possesses two ClpP paralogs—ClpP1 and ClpP2—that form a functional heterotetradecamer consisting of two stacked heptameric rings (ClpP1P2) (7). A recent study has found that only ClpP1 confers the essential proteolytic activity to the complex, while ClpP2 facilitates chaperone binding (8). In Mtb, two distinct Clp ATPases, ClpC1 or ClpX, can interact with the ClpP2 subunit, recognizing, unfolding, and delivering their respective potential substrate proteins into the ClpP1P2 proteolytic chamber for degradation (9).

Moreover, unlike other bacteria, the genes encoding all components of the Mtb Clp are essential. Precisely for this reason, these genes are regarded as highly promising therapeutic targets for anti-tuberculosis drug development (10, 11). Several compounds targeting Mtb ClpP1P2 have been identified. For example, the bortezomib analog Pyr-FL-CMK and β-lactone derivatives such as EZ120 display selective inhibitory activity against Mtb ClpP (12, 13), while acyldepsipeptides (ADEPs) inhibit Mtb growth by activating Mtb ClpP1P2 proteolysis (14). Cediranib and its derivatives have also been identified as potent Mtb ClpP1P2 inhibitors targeting a unique allosteric site (15). However, it is noteworthy that the hydrophobic clefts targeted by ADEPs in Mtb ClpP1P2 are conserved in human mitochondrial ClpP, and ADEP binding can lead to mitochondrial dysfunction (16). This finding raises concerns that highly conserved protein subunits within Clps may not be ideal targets for the development of next-generation anti-TB drugs. To date, few inhibitors or activators directly targeting the ClpP1P2 chaperone ClpX have been reported. Recently, one study demonstrated that ilamycin E can disrupt ClpC1P1P2-mediated proteolysis and also inhibit the proteolytic activity of ClpXP1P2 in a manner dependent on the substrate’s tag sequence and adaptor (17). Notably, ClpC1, unlike ClpX, lacks animal homologs, making it an especially attractive target for modulating Mtb Clp activity. The ribosomally synthesized cyclic peptide lassomycin and the macrocyclic tridecapeptide ecumicin (Ecu) both kill Mtb by targeting the ClpC1 ATPase complex (18, 19); additionally, cyclomarin A acts by excessively activating ClpC1, increasing the ClpC1-mediated turnover of cellular proteins and thereby interfering with the normal function of ClpPs (20). These cyclic peptides have exhibited potent antibacterial activity against both MDR and XDR Mtb, with ecumicin shown to be effective at killing Mtb in both macrophages and murine models (21). Although the pharmacodynamic and pharmacokinetic properties of these compounds still require optimization, they represent promising new avenues in the fight against Mtb.

In Mtb, the degradation of damaged proteins by Clps is tightly regulated through precise recognition by AAA+ partners and substrate availability (22). Consequently, most studies have concentrated on the substrate spectrum of the complex. Following the controlled knockdown of *clpP1P2* under non-stress conditions, over 100 candidate substrates have been identified (23). Recent studies indicated that 85% of the candidate protein sequences recognized by ClpC1P1P2 are rich in terminal disordered regions (24). The identified substrates are involved in a wide range of essential cellular functions, including central metabolism, stress regulation, and pathogenicity of Mtb (12, 25, 26). This underscores the crucial role of Clps not only in general proteostasis but also in stress response and virulence regulation.

While significant attention has been paid to Clp substrate recognition and the discovery of functional asymmetry between ClpP1 and ClpP2 (27), it is notable that mycobacteria possess non-redundant unfoldases, ClpC1 and ClpX (9). To our knowledge, no studies have directly compared their specific physiological functions. It has been reported that ClpX activity is regulated by single-stranded DNA-binding protein and that ClpX plays an important role during the DNA replication phase of the mycobacterial cell cycle (28). However, the precise physiological roles and functional breadth of ClpC1 in mycobacteria remain to be elucidated.

In this study, we found that ClpX impairment in *Mycobacterium smegmatis* and the attenuated strain *Mycobacterium tuberculosis* H37Ra disrupts core cellular metabolism, including the fatty acid pathway, membrane lipid systems, and overall proteostasis. In contrast, ClpC1 dysfunction in these non-pathogenic mycobacterial models specifically compromised mycobacterial iron homeostasis, triggering the Fenton-mediated lethal oxidative stress under oxygen exposure. Given that, during TB reactivation, the environment transitions from hypoxic to oxygen-rich, the ability to adapt to fluctuating oxygen levels is thought to influence survival during shifts between latent and active states. Our results highlight a possible role for ClpC1 in maintaining the cellular iron pool homeostasis in mycobacteria, thereby providing insight into mechanisms relevant to oxidative stress management during oxygen exposure. This finding advances our understanding of Clp function in mycobacterial physiology, and this discovery may inform future therapeutic approaches that specifically target the reactivation phase of latent TB.

## MATERIALS AND METHODS

### Strains, plasmids, and culture conditions

*Mycolicibacterium smegmatis* (Msm) MC² 155 and Mtb H37Ra were maintained in the laboratory. *Escherichia coli* (DH5α) strain was from Sangon Biotech. Plasmids PLJR965 and PLJR962 were generously provided by the Sarah M. Fortune Lab at Harvard University. Msm and Mtb were cultured in Middlebrook 7H9 or 7H10 broth (BD) supplemented with 0.2% glycerol, 0.05% Tween 80, and 10% OADC (0.5% bovine albumin fraction V, 0.085% NaCl, 0.0004% catalase, 0.2% glucose, 0.012% oleic acid), with shaking to mid-logphase (OD_600_ = 0.3–0.6). Where indicated, 100 ng/mL ATC, 50 µg/mL kanamycin, and 150 µg/mL hygromycin were added to maintain plasmid selection or gene knockdown. For specific stress, redox susceptibility, and iron chelation assays, cultures were supplemented with exogenous catalase (30 µg/mL), dithiothreitol (DTT, 1 mM), 4-hydroxy-TEMPO (4HT, 1 mM), N-acetyl-L-cysteine (NAC, 2 mM), or deferoxamine (DFO, 100 µg/mL), as indicated. For HDO conditions, a 250 mL Erlenmeyer flask with a gauze cap was used instead of a screw-cap bottle to allow continuous aeration and diffusion of oxygen from the atmosphere. For iron-depletion studies, iron-free Roisin’s medium was used. Carbon dioxide gas packs (AnaeroPack, Mitsubishi) were employed to create an anaerobic environment.

### Construction of knockdown strains

To construct knockdown strains, we employed the Clustered Regularly Interspaced Short Palindromic Repeats (CRISPR) dCas9 method (29). Specifically, sgRNAs were designed according to PAM site rules and cloned into PLJR962 or PLJR965 plasmids. The plasmids were then transformed into *E. coli* DH5α competent cells by heat shock and grown on Luria-Bertani (LB) agar plates with kanamycin (50 µg/mL). Correctly sequenced plasmids were electroporated into Msm and Mtb competent cells under conditions of 1,000 Ω, 2.5 kV, and 25 µF, and then were grown on 7H10-OADC agar with kanamycin (30 µg/mL). Single colonies were selected and grown in the presence of 100 ng/mL ATC until reaching the log-phase. The knockdown efficiency of specific genes was determined by real-time PCR.

### Detection of transcription levels under different stress conditions

Mtb was inoculated into 5 mL of 7H9-OADC medium at 2% and incubated at 37°C with shaking at 100 rpm until OD_600_ ~0.6. The culture was then transferred to liquid medium at a 0.5% ratio and grown to the log-phase. Bacteria were collected and resuspended in 7H9 medium at pH 7 and pH 5 to represent normal and acidic conditions, respectively. For starvation conditions, bacteria were resuspended in sterile PBS. For hypoxia, we employed AnaeroPack to create an anaerobic environment. All cultures were incubated at 37°C, with acidic and starvation conditions maintained for 4 h and 24 h, respectively, and hypoxic conditions for 4 h and 48 h. After treatment, specific genes were analyzed by RT-qPCR.

### RT-qPCR

Total RNA extraction was performed using the TRIzol method (Invitrogen, USA). One microgram of RNA was reverse transcribed into cDNA using the HiScript II One-Step RT-PCR Kit (Dye Plus) (Vazyme, China). Each reaction was amplified in triplicate with Taq-Pro Universal SYBR qPCR Master Mix (Vazyme, China). Relative gene expression was normalized to sigA and calculated via the 2^(−ΔΔCt)^ method.

### Growth curve measurement

Strains were inoculated into different media and grown to the log-phase. The bacterial cultures were then diluted 1:100 into 50 mL of the corresponding medium and incubated at 37°C with shaking at 100 rpm. OD_600_ was measured at 0 h and every 2 h thereafter for up to 24 h. Each experiment was performed in triplicate.

### Bacterial spot plating assay

Bacterial suspensions were adjusted to OD_600_ ~0.1 and subjected to 10-fold serial dilution. Aliquots (10 μL) from each dilution series were spotted onto different agar plates supplemented with ATC. Plates were incubated at 37°C for 4 weeks (Mtb) or 4 days (Msm) before analysis, with three biological replicates per experimental group.

### Determination of dissolved oxygen concentration

Samples were collected in specialized dissolved oxygen bottles, filled via siphoning with overflow to eliminate air bubbles, and dissolved oxygen was fixed immediately on-site by injecting 1 mL of manganous sulfate solution and 2 mL of alkaline iodide solution below the liquid surface. The bottle was sealed and inverted repeatedly for mixing, followed by sedimentation of the precipitate. Subsequently, 2 mL of concentrated sulfuric acid was injected below the liquid surface. The mixture was vigorously shaken until complete dissolution of the precipitate and stored in the dark for 5 min to liberate free iodine. A 100 mL aliquot of the treated solution was transferred to a conical flask and titrated with sodium thiosulfate standard solution until pale yellow. Then, 1 mL of starch indicator was added, and titration continued until the blue color disappeared entirely. The volume of sodium thiosulfate consumed was recorded for dissolved oxygen concentration calculation.

### Transmission electron microscopy imaging

Bacteria were harvested by centrifugation, washed three times with sterile PBS, and fixed with 2% paraformaldehyde and 2.5% glutaraldehyde for 15 min. After washing three times with ddH_2_O, 2.5 µL of the bacterial suspension was placed on a 200-mesh carbon-coated grid and stained with 2.5 µL of 5% uranyl acetate and 3% lead citrate. After drying, the bacteria were observed and photographed using a transmission electron microscope (Thermo).

### Cell infection

Log-phase bacterial suspensions were prepared and used to infect THP-1 cells (ATCC), which had been differentiated with 10 nM phorbol 12-myristate 13-acetate for 48 h in RPMI 1640 medium. Infections were carried out at a multiplicity of infection of 10. At designated time points post-infection, the cells were lysed with sodium dodecyl sulfate (SDS), and viable bacteria were quantified by CFU counting. Each condition was tested in three biological replicates.

### Reactive oxygen species (ROS) content measurement in different conditions

After bacterial cultures reached the log-phase under different conditions, they were resuspended in fresh medium and adjusted to OD_600_ ~0.2. Ten micromolar DHE (dihydroethidium) was added, and 200 µL of the bacterial suspension was placed in a black 96-well plate, with three replicates set for each group. After incubation at 37°C for 30 min, fluorescence intensity was measured using a fluorescence microplate reader with excitation/emission wavelengths of 518/605 nm. Cumene hydroperoxide (15 mM) was used as a positive control, and CFU counting was performed on the OD_600_ ~0.2 bacterial suspension to calculate relative ROS levels.

### Measurement of intracellular iron content

Following sample lysis and protein quantification, intracellular iron levels were determined using two specifically formulated reagents: The total iron detection reagent contained ferrozine (0.32 g), neocuproine (0.135 g), ammonium acetate (19.25 g), and ascorbic acid (17.6 g) in deionized water (final volume 100 mL), while the free ferrous iron detection reagent used identical components except for ascorbic acid. For quantification, 100 μL of lysate was combined with 100 μL PBS and 30 μL total iron reagent for total iron measurement, or with 30 μL free ferrous iron reagent for free ferrous iron detection; all mixtures were incubated at 37°C for 15 min before measuring OD_550_.

### TUNEL DNA strand breaks analysis

DNA fragmentation was quantified using the TUNEL assay (Click-iT Plus TUNEL Kit, Thermo Fisher) per manufacturer’s protocol, with flow cytometry analysis (BD FACSCalibur) detecting FITC-labeled DNA breaks. Three replicates were analyzed for each group.

### MIC determination

Msm strains were grown to log-phase in different media and adjusted to an OD_600_ ~0.1. In a 96-well cell culture plate, 100 μL of bacterial suspension was added to the first column of wells, along with the highest concentration of the drug. Serial two-fold dilutions were performed in the subsequent wells. Each bacterial strain was tested in triplicate. The plate was incubated at 37°C for approximately 5 days, and the MIC was determined as the lowest drug concentration that prevented visible growth of the bacteria. For the Ecu and DTT combination test, a fixed concentration of DTT (0.0625 mg/mL) was added to the wells, and Ecu was serially diluted.

### Antimicrobial activity assay

Msm was cultured to the log-phase and then resuspended in fresh 7H9-OADC medium to an OD_600_ ~0.2. The bacteria were treated with the corresponding drug combinations: rifampicin (RIF) at a final concentration of 20 μg/mL, isoniazid (INH) at 40 μg/mL, and ciprofloxacin (CIP) at 20 μg/mL. Cultures were incubated at 37°C, and CFU counts were performed on different days. All experiments were performed in triplicate.

### Proteomics analysis

When bacteria reached the log-phase, they were harvested by centrifugation, washed three times with sterile PBS, and transferred to 2 mL cryovials. The samples were snap-frozen in liquid nitrogen for 1 min and stored at −80°C until further processing. Three independent biological replicates were prepared for each experimental group. Proteomics analysis was performed by sending the samples to Bioprofile Co., Ltd. (China) for TMT-based quantification (MS2 level). Protein identifications were filtered at a 1% false discovery rate. Differentially expressed proteins were screened with the cutoff of a fold-change ratio of ≥1.50 or ≤0.67 and *P* values <0.05, and the results were analyzed using clustering, heat maps, Gene Ontology (GO) analysis, and KEGG analysis mapping.

### ATP content measurement

Bacteria from different strains were grown to the log-phase, and 1 mL of culture was collected and centrifuged. The supernatant was discarded, and the pellet was washed three times. The bacterial suspension was transferred to cryovials containing glass beads and disrupted using a vortex mixer with 5 cycles of 1 min each, with 1 min of incubation on ice between cycles. After centrifugation, the supernatant was used to determine protein concentration by the BCA assay. ATP content was measured using the ATP assay kit (Promega), with three replicates per experiment, and relative ATP content was calculated based on protein concentration.

### Determination of intracellular ergothioneine (EGT) content by LC-MS

Two milliliters of bacterial culture grown to log-phase was collected, washed twice with sterile PBS, and then resuspended in 2 mL of 70% acetonitrile containing 25 ng/mL of the internal standard 1-methyl-4-phenylpyridinium (MPP+). The resuspended bacterial suspension was transferred to a cryovial containing glass beads and subjected to a tissue disruptor. Parameters were set to 40 Hz for 30 s, followed by cooling at −80°C for 1 min. This disruption step was repeated five times. The sample was then stored in a dry ice container until analysis. Prior to analysis, the bacterial suspension was filtered through a 0.22 μm filter. LC-MS conditions were as follows: solvent (76% acetonitrile, 24% water, 0.1% formic acid), flow rate 200 μL/min. EGT and MPP+ parameters were: EGT (m/z parent, m/z fragment, and CE): 230, 127, and 27 V; MPP+ (m/z parent, m/z fragment, and CE): 171, 129, and 45 V. Each bacterial strain was tested in triplicate.

### Measurement of trehalose content

Samples were prepared following the method used for ATP measurement. Trehalose content was measured using the Trehalose Assay Kit (Megazyme) in strict adherence to the manufacturer’s instructions, with each experiment performed in triplicate.

### Construction of overexpression strains

To distinguish the resistance of overexpression plasmids from knockdown plasmids, we removed the kanamycin resistance gene from the overexpression plasmid and replaced it with an amplified hygromycin resistance gene, converting the overexpression plasmid PMV261 from kanamycin resistance to hygromycin resistance. We then PCR-amplified the overexpression target gene with a His-tag and cloned it into the PMV261-hyg plasmid. The plasmid was transformed into *E. coli* DH5α competent cells by heat shock and grown on LB agar with hygromycin (225 µg/mL). Correctly sequenced plasmids were electroporated into the *clpC1* knockdown strain competent cells under the same conditions and grown on 7H10-OADC agar with kanamycin (30 µg/mL) and hygromycin (150 μg/mL). Single colonies were selected, and the knockdown strains were grown in the presence of 100 ng/mL ATC.

### Western blot

Standard Western blotting techniques were employed. Briefly, 20 μg of bacterial protein extracts were separated on a 12.5% SDS-PAGE gel and transferred to a PVDF membrane. The membrane was blocked with 5% non-fat milk at room temperature for 2 h, followed by overnight incubation at 4°C with the corresponding antibody diluted at 1:5,000. The ICD2 protein was used as a loading control. The membrane was washed three times with TBST, incubated with the secondary antibody (1:5,000), and washed again three times. Signal detection was performed using an enhanced chemiluminescence reagent kit.

### Mice infection

C57BL/6 mice were fed a doxycycline-containing diet starting 3 days prior to infection to induce gene knockdown. For iron modulation experiments, mice received a single intraperitoneal (i.p.) injection of iron dextran (FeDex, 4 mg/mouse) on day −1, or daily i.p. injections of deferoxamine (DFO, 100 mg/kg) from day −1 to day 12. Control groups received an equivalent volume of PBS vehicle. On day 0, mice were anesthetized and infected intranasally with 10⁷ CFU of Msm strains suspended in 40 μL PBS. At 1, 4, 8, and 12 days post-infection, lungs were harvested and homogenized for bacterial burden determination (CFU counting). For histopathological analysis, lung tissues were fixed in 4% paraformaldehyde and stained with hematoxylin and eosin (H&E).

### Statistical analysis

All experiments were performed with at least three independent biological replicates unless otherwise stated. Statistical analyses were conducted using GraphPad Prism 9.0 (GraphPad Software, USA). Data are presented as mean ± standard deviation (SD) unless indicated. Two-tailed unpaired Student’s *t*-tests were used for pairwise comparisons, while one-way analysis of variance (ANOVA) with Tukey’s *post hoc* test was applied for comparisons among multiple groups. Prior to applying ANOVA, normal distribution was verified using the Shapiro-Wilk test (or assumed based on standard biological distribution for small sample sizes), and homogeneity of variance was confirmed using the Brown-Forsythe test. A *P* value of <0.05 was considered statistically significant (ns, not significant; *P < 0.05, **P < 0.01, ***P < 0.001, ****P < 0.0001). No data points were excluded, and all experiments were repeated independently at least three times to ensure reproducibility.

## RESULTS

### Mycobacterial ClpC1 and ClpX subunits exhibit a functional division of labor under environmental stress, governing distinct growth and adaptive phenotypes

To determine whether ClpX and ClpC1 exhibit functional specialization in Mtb, we analyzed their expression in the attenuated Mtb H37Ra strain under various stresses. Hypoxia induced rapid *clpC1* upregulation but downregulated *clpX* by 39% at 48 h. Acid stress (pH 5.0) upregulated both genes (*clpC1*: 6.0-fold; *clpX*: 1.8-fold), while nutrient starvation specifically increased *clpC1* by 3.0-fold (Fig. S1).

**Fig 1:**
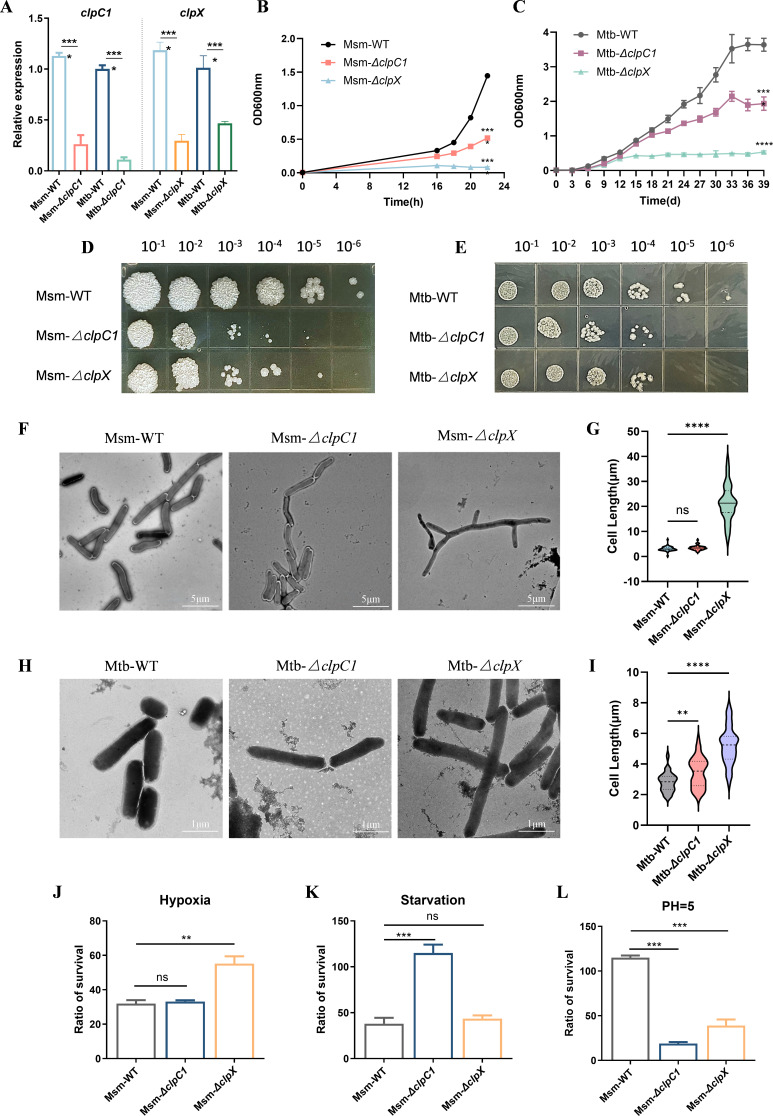
Growth, morphological features, and survival abilities of *clpC1* and *clpX* knockdown strains in Msm and Mtb. (**A**) Validation of *clpC1* and *clpX* mRNA knockdown in different strains using RT-qPCR. Gene expression levels were normalized to the housekeeping gene sigA. (**B and C**) Growth curves of WT, *clpC1*- and *clpX*-knockdown strains of Msm (**B**) and Mtb (**C**) cultured in 7H9-OADC medium, monitored by measuring OD₆₀₀ over time. (**D and E**) Colony formation of WT and knockdown strains on 7H10-OADC agar plates, with 10-fold serial dilutions of bacterial suspensions for Msm (**D**) and Mtb (**E**). (**F and H**) Representative electron microscopy images depicting cell morphology of Msm (**F**) and Mtb (**H**) strains. (**G and I**) Quantitative analysis of cell length in Msm (**G**) and Mtb (**I**) strains based on electron microscopy images. (**J–L**) Survival ratios of WT and knockdown strains after 48 h exposure to hypoxia (**J**), nutrient starvation (**K**), and low pH (**L**) in 7H9-OADC medium. Data are presented as mean ± SD from three biological replicates. Statistical significance was determined by one-way ANOVA followed by Tukey’s multiple comparison test (*P < 0.05; **P < 0.01; ***P < 0.001; ****P < 0.0001; ns, not significant).

Given the essentiality of *clpC1* and *clpX*, we employed a CRISPR interference system to generate conditional knockdown strains in both Msm (Msm-Δ*clpC1* and Msm-Δ*clpX*) and Mtb (Mtb-Δ*clpC1* and Mtb-Δ*clpX*) (29). Following anhydrotetracycline (ATC) induction, transcription of *clpC1* and *clpX* was significantly suppressed (Fig. 1A). To rule out potential polar effects, we confirmed via RT-qPCR that the expression of adjacent downstream genes remained unaffected following *clpC1* or *clpX* knockdown in both Msm and Mtb (Fig. S2). Compared to WT strains, knockdown of either *clpC1* or *clpX* impaired bacterial growth in both species (Fig. 1B through E). Notably, in 7H9-OADC liquid medium, the growth of both Msm-Δ*clpX* and Mtb-Δ*clpX* was nearly completely arrested, whereas the *clpC1* knockdown strains exhibited only moderately slower growth. Transmission electron microscopy showed pronounced morphological abnormalities, including filamentation and branching in Msm-Δ*clpX*, with approximately 92% of cells exceeding 10 μm in length (Fig. 1F and G), and Mtb-Δ*clpX* showing a 1.85-fold increase (Fig. 1H and I). This filamentous and branching cell death phenotype is consistent with previous reports of strains lacking the Mtb FtsZ and a ClpX target protein essential for cell division (28, 30). By contrast, Mtb-Δ*clpC1* and Msm-Δ*clpC1* strains exhibited only moderate or no elongation.

Stress tolerance assays also revealed distinct phenotypic differences. Under hypoxia, Msm-Δ*clpX* survival increased to ~55% versus ~32% for WT and Msm-Δ*clpC1* (Fig. 1J). Conversely, under nutrient starvation, Msm-Δ*clpC1* survival rose to ~115%, while Msm-Δ*clpX* was unaffected (Fig. 1K). Both mutants were acid-sensitive, with survival rates of 18% (Msm-Δ*clpC1*) and 39% (Msm-Δ*clpX*) at pH 5.0 (Fig. 1L). In THP-1 macrophages, *clpX* knockdown in Msm and Mtb both exhibited significant survival defects (reduction of 1.88 log_10_ and up to 1.61 log_10_ CFUs, respectively), whereas *clpC1* knockdown had no significant effect on the intracellular survival of either Msm or Mtb at 24 h post-infection (Fig. S3A and B). Collectively, these results show that *clpX* knockdown exerts a more severe impact on fundamental growth and division than does ClpC1 depletion, while ClpC1 and ClpX play distinct, non-overlapping roles in stress adaptation in mycobacteria.

### ClpC1-depleted mycobacteria are hypersensitive to oxygen exposure and exogenous catalase

Notably, compared to the growth in 7H9-OADC liquid medium, the *clpC1* knockdown strains displayed slower growth on 7H10-OADC agar plates, even more so than the *clpX* knockdown strains, in both Msm-Δ*clpC1* and Mtb-Δ*clpC1* (Fig. 1D and E). During mutant characterization, liquid cultures were grown in sealed screw-cap flasks shaken at 50 rpm (headspace/media ratio of 5:1), minimizing atmospheric oxygen diffusion. In contrast, growth on agar plates in petri dishes allowed continuous aeration and thus sufficient oxygen exposure. This led us to hypothesize that *clpC1* knockdown strains are hypersensitive to elevated oxygen levels.

**Fig 2:**
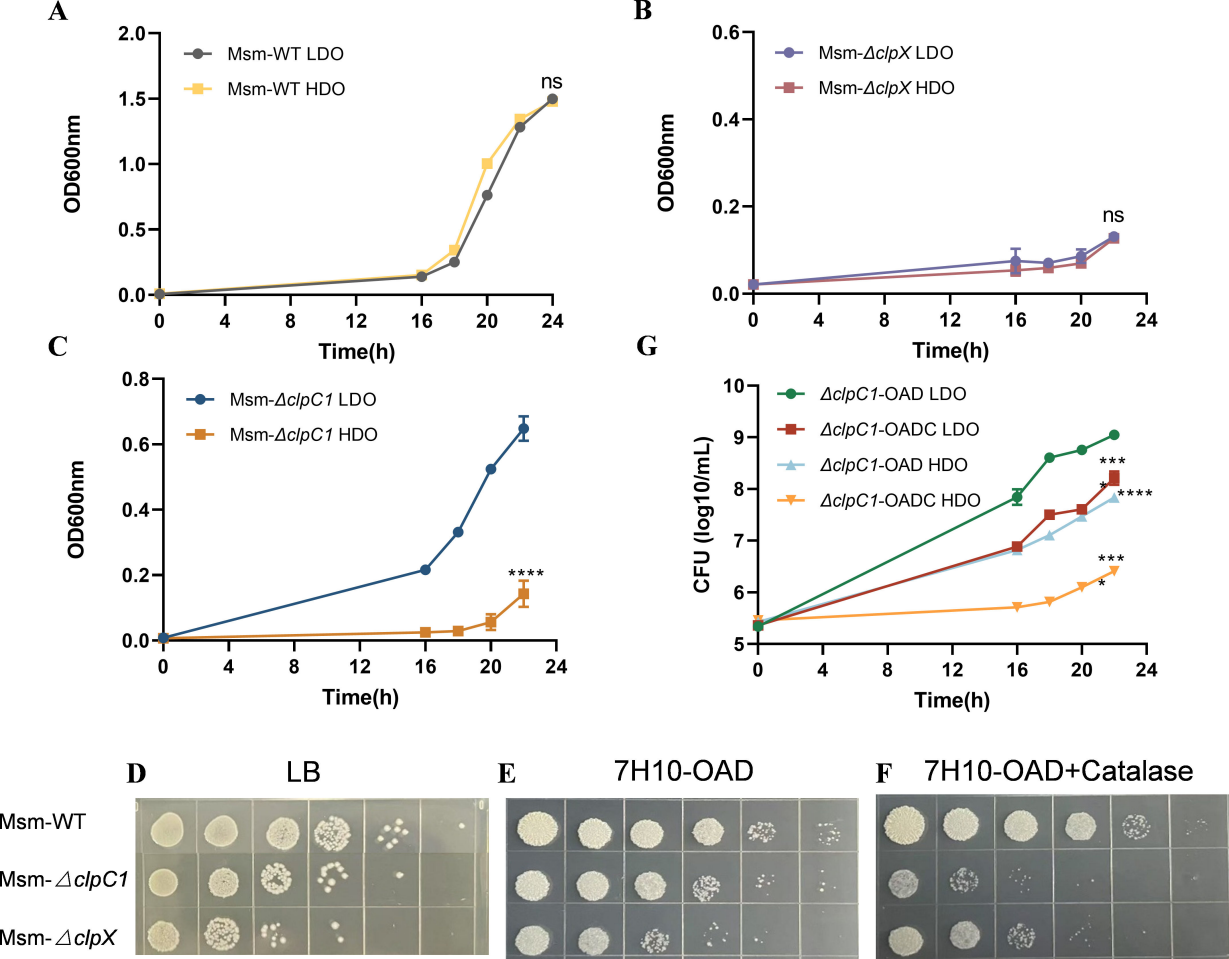
The *clpC1* knockdown strain is sensitive to high dissolved oxygen and catalase. (**A–C**) Growth curves of Msm-WT (**A**), Msm-Δ*clpX* (**B**), and Msm-Δ*clpC1* (**C**) under HDO and LDO conditions in 7H9-OADC medium. (**D–F**) Colony formation of Msm-WT, Msm-Δ*clpC1*, and Msm-Δ*clpX* plated on LB (**D**), 7H10-OAD (**E**), and 7H10-OAD supplemented with catalase (**F**) agar with 10-fold serial dilutions of each culture. (**G**) Growth kinetics of Msm-Δ*clpC1* in 7H9 medium under HDO and LDO conditions in the presence (OADC) or absence (OAD) of catalase, as determined by CFU assays. Data represent mean ± SD from three independent biological replicates. Statistical significance was evaluated by one-way ANOVA with Tukey’s multiple comparison test (***P < 0.001; ****P < 0.0001; ns, not significant).

To test this, we compared the growth of WT, Msm-Δ*clpC1*, and Msm-Δ*clpX* in 7H9-OADC liquid medium using either sealed flasks (low dissolved oxygen; LDO) or oxygen-permeable caps (high dissolved oxygen; HDO). The WT strain grew similarly under both conditions (Fig. 2A), and Msm-Δ*clpX* remained growth arrested (Fig. 2B). In sharp contrast, growth of Msm-Δ*clpC1* was severely compromised under HDO conditions (Fig. 2C). Direct measurements showed average dissolved oxygen concentrations of 6.61 mg/L for HDO and 2.41 mg/L for LDO in Msm-Δ*clpC1* cultures after 20 h (given that at 37°C, the maximum dissolved oxygen in water is ~6.7 mg/L) (31). These results indicate that ClpC1-deficient strains are extremely sensitive to high dissolved oxygen exposure.

High dissolved oxygen increases cellular ROS, impairing bacterial growth (32). Catalase is routinely supplemented in culture medium as a ROS scavenger. We hypothesized that in the absence of catalase, *clpC1* knockdown strains would have especially low survival under aerating conditions. We evaluated growth on LB agar medium and 7H10 medium with either OADC (with catalase) or OAD (without catalase). Msm-Δ*clpC1* grew slower than WT but faster than Msm-Δ*clpX* on both LB and 7H10-OAD agar (Fig. 2D and E). Unexpectedly, omission of catalase from 7H10-OAD medium did not further slow down Msm-Δ*clpC1* growth (Fig. 2E); conversely, the presence of catalase in agar medium significantly decreased its viability (Fig. 2F).

To further evaluate this phenomenon, we monitored the growth of Msm-Δ*clpC1* under HDO and LDO conditions in 7H9-OAD liquid medium with or without catalase, respectively. ClpC1-deficient strains demonstrated heightened sensitivity under HDO regardless of catalase presence. Notably, supplementation with catalase in HDO conditions caused a 2.13 log_10_ (*P* < 0.001) reduction in CFU after 16 h compared to LDO (Fig. 2G). Even under LDO, catalase addition inhibited Msm-Δ*clpC1* growth, resulting in a 1.05 log_10_ reduction in CFU after 16 h (*P* < 0.001), whereas this effect was absent in catalase-free medium (Fig. 2G). Collectively, these results indicate that ClpC1 impairment significantly reduces resilience to high dissolved oxygen. Moreover, the presence of catalase dramatically exacerbates the sensitivity of Msm-Δ*clpC1* to dissolved oxygen, even preventing bacterial growth under normal atmospheric oxygen levels. This suggests that ClpC1 is likely a key regulatory component in the mycobacterial response to oxygen exposure.

**Fig 3:**
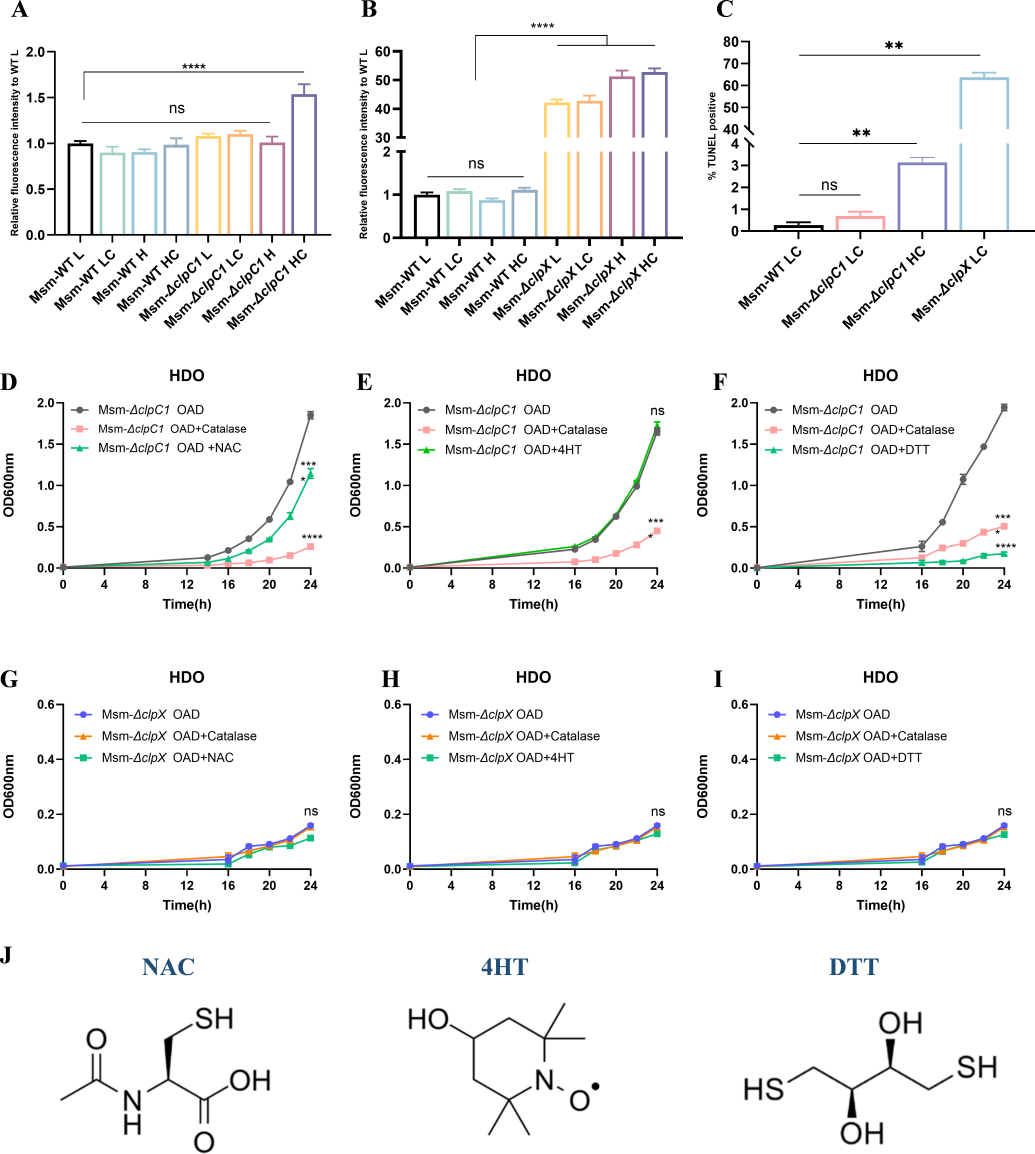
Sulfhydryl reductants specifically exacerbate oxidative damage in Msm-Δ*clpC1*. (**A and B**) Measurement of intracellular ROS levels in Msm-Δ*clpC1* (**A**) and Msm-Δ*clpX* (**B**) strains under four conditions: LDO (L), LDO with catalase (LC), HDO (H), and HDO with catalase (HC). (**C**) Quantification of DNA damage in Msm-WT, Msm-Δ*clpC1*, and Msm-Δ*clpX* under various oxygen and catalase conditions. (**D–F**) Growth curves of Msm-Δ*clpC1* cultured in 7H9-OAD medium under HDO, with or without supplementation with catalase, NAC, 4HT, and DTT, as indicated. (**G–I**) Corresponding growth curves of Msm-Δ*clpX* under the same conditions. (**J**) Chemical structures and functional groups of the three reductants (NAC, 4HT, and DTT). Data are shown as mean ± SD from three independent biological replicates. Statistical analysis was performed using one-way ANOVA with Tukey’s multiple comparison test (*P < 0.05; **P < 0.01; ****P < 0.0001; ns, not significant).

We further compared total ROS levels in Msm-Δ*clpC1* and Msm-Δ*clpX* grown under various dissolved oxygen conditions using 7H9-OADC and 7H9-OAD media. While *clpC1* knockdown alone did not affect intracellular ROS, it caused a significant increase under HDO with catalase (Fig. 3A). In contrast, Msm-Δ*clpX* showed pronounced ROS accumulation under all tested conditions, even exceeding 40-fold (Fig. 3B). Since EGT helps maintain redox homeostasis in mycobacteria (33), we measured its levels using LC-MS. EGT was reduced to 39% of WT in Msm-Δ*clpC1* and 54% in Msm-Δ*clpX* (Fig. S4).

Excessive ROS can directly cause DNA damage (34, 35), and DNA damage was further quantified using TUNEL assays. After 2 days of growth in 7H9-OADC medium under LDO conditions, Msm-Δ*clpX* showed 60% more DNA damage than WT, while Msm-Δ*clpC1* showed no significant difference. Strikingly, under HDO with catalase, DNA damage in Msm-Δ*clpC1* increased over 4-fold compared to LDO (Fig. 3C). These findings suggest that loss of ClpC1 and ClpX impairs redox homeostasis through distinct mechanisms: ClpX loss directly disrupts redox balance, leading to ROS accumulation and DNA damage, whereas ClpC1 may regulate redox homeostasis by sensing or responding to environmental oxygen levels.

### Iron dysregulation drives Fenton-mediated lethality in ClpC1-deficient mycobacteria and dictates infection outcomes

Supplementation with exogenous antioxidants not only severely inhibited the growth of Msm-Δ*clpC1* (Fig. 2G), but also promoted the accumulation of ROS (Fig. 3A), revealing a counterintuitive phenomenon. Therefore, we further investigate whether other antioxidants, such as NAC (36) and 4HT (37), exert similar inhibitory effects to catalase on *clpC1* knockdown strains. Intriguingly, NAC exacerbated growth inhibition in oxygen-replete cultures of Msm-Δ*clpC1* (Fig. 3D). However, the addition of 4HT did not elicit this effect (Fig. 3E). Given the thiol (-SH) group in NAC, we tested DTT, a dithiol-containing reducing agent (Fig. 3J). DTT similarly intensified growth suppression in Msm-Δ*clpC1* (Fig. 3F), suggesting that thiol groups paradoxically aggravate stress with ClpC1 deficiency. The same conditions were applied to Msm-Δ*clpX*. The results showed that neither NAC (Fig. 3G), 4HT (Fig. 3H), nor DTT (Fig. 3I) could alleviate the growth defect of Msm-Δ*clpX*.

**Fig 4:**
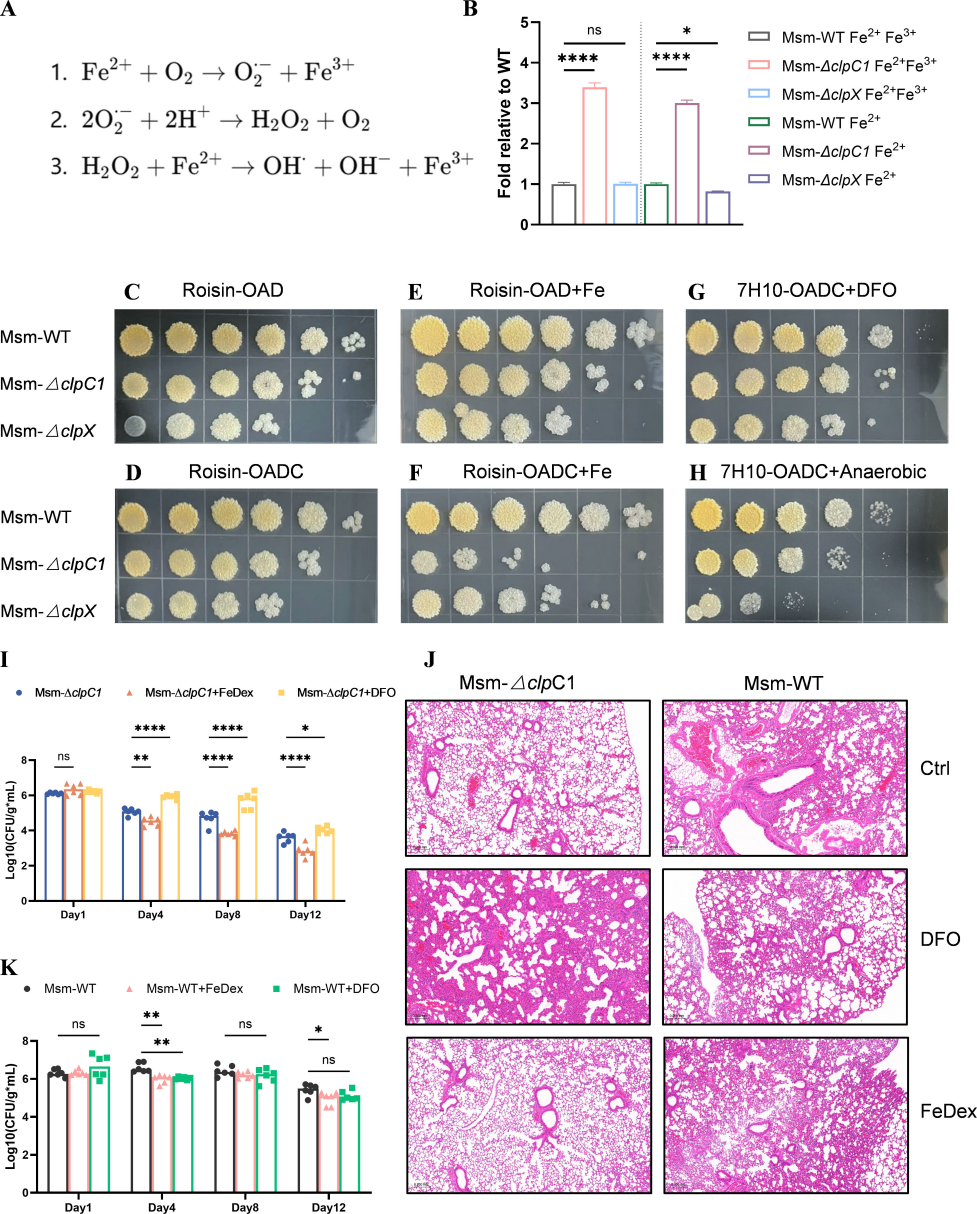
Disrupted iron homeostasis determines the susceptibility of ClpC1-deficient mycobacteria to host iron availability. (**A**) The three steps of the Fenton reaction. (**B**) Levels of bivalent (Fe²^+^) and trivalent (Fe³^+^) iron ions in Msm-WT, Msm-Δ*clpC1*, and Msm-Δ*clpX* strains in 7H9-OADC medium. (**C–H**) Growth observation of Msm-WT, Msm-Δ*clpC1*, and Msm-Δ*clpX* on different agar plates under the indicated conditions. Bacterial burdens of Msm-Δ*clpC1* (**I**) and Msm-WT (**K**) in C57BL/6 mice lungs treated with FeDex (iron dextran) or DFO compared to the PBS control group. (**J**) Representative H&E staining images of lung tissues collected at day 8 post-infection from Msm-Δ*clpC1*- and Msm-WT-infected mice under FeDex or DFO treatment. Data represent mean ± SD from three independent biological replicates. Statistical significance was determined by one-way ANOVA followed by Tukey’s multiple comparison test (*P < 0.05; **P < 0.01; ****P < 0.0001; ns, not significant).

This paradoxical finding suggests that the reducing properties of sulfhydryl compounds further promote ROS accumulation and sensitize ClpC1-deficient strains to oxygen exposure. This phenomenon is likely driven by an endogenous Fenton cascade triggered in the presence of oxygen and reducing agents. The Fenton reaction, together with the Haber-Weiss cycle, acts synergistically on intracellular iron, leading to a rapid generation of highly reactive species, particularly hydroxyl radicals (35). The process involves three key steps: (i) Ferrous iron (Fe²^+^) reacts with oxygen to produce superoxide radicals (O_2_·⁻); (ii) Superoxide radicals undergo dismutation to form hydrogen peroxide (H_2_O_2_); (iii) H_2_O_2_ reacts with Fe²^+^ via the Fenton reaction, generating hydroxyl radicals (·OH) (Fig. 4A). This cascade requires both oxygen and Fe²^+^, suggesting that ClpC1 deficiency may disrupt iron metabolism in mycobacteria. Consistent with this hypothesis, measurements of intracellular iron revealed that Msm-Δ*clpC1* accumulated more than three times as much Fe²^+^ as the control strains, whereas Fe²^+^ levels remained unchanged in Msm-Δ*clpX* (Fig. 4B). Similarly, Mtb-Δ*clpC1* also exhibited significant iron accumulation (Fig. S5A), highlighting the unique role of ClpC1 in iron regulation. Catalase uses heme (iron) as a cofactor to decompose H_2_O_2_ into water and oxygen (2H_2_O_2_ → 2H_2_O + O_2_) rapidly (38). We hypothesize that in the high-Fe²^+^ environment characteristic of Msm-Δ*clpC1*, the additional O_2_ produced by catalase activity is no longer benign. Instead, this excess O_2_ fuels the initial step of the Fenton cascade (Fe²^+^ + O_2_ → O_2_·⁻), further aggravating the growth impairment of Msm-Δ*clpC1*. Phenotypic analysis across various culture media further supported this hypothesis: on iron-deficient Roisin agar plates, the growth of Msm-Δ*clpC1* was unaffected by the presence or absence of catalase (Fig. 4C and D), whereas supplementation with exogenous iron resulted in a pronounced, catalase-dependent growth defect (Fig. 4E and F). Furthermore, both deferoxamine treatment (an iron chelator) (Fig. 4G) and anaerobic cultivation (Fig. 4H) alleviated the growth inhibition of Msm-Δ*clpC1*. Notably, this iron-dependent oxidative vulnerability was not observed in Msm-Δ*clpX* (Fig. 4C through G). These results indicate that the combination of iron ions and oxygen promotes the Fenton reaction, leading to the severe growth defects specific to Msm-Δ*clpC1*, and any factor that enhances this reaction, such as oxygen exposure, catalase, or the presence of sulfhydryl compounds, can exacerbate the phenotype. This suggests that ClpC1 (rather than ClpX) plays a critical role in maintaining intracellular iron pool homeostasis and safeguarding the bacteria against oxidative damage upon oxygen exposure.

To validate whether this iron-dependent vulnerability drives reduced survival *in vivo*, we infected mice with the respective strains. Consistent with their *in vitro* growth defects, the Msm-Δ*clpX* strain was rapidly cleared from the lungs, while Msm-Δ*clpC1* exhibited an intermediate survival defect compared to WT (Fig. S5B). Histological analysis paralleled these findings: Msm-Δ*clpX* induced minimal lung injury, whereas Msm-Δ*clpC1* caused intermediate pathology compared to the severe inflammation observed in wild-type infections (Fig. S5C).

Given that iron availability dictates ClpC1-deficient bacterial survival *in vitro*, we next tested whether modulating host iron levels would similarly impact infection outcomes *in vivo*. Iron supplementation with iron dextran (FeDex) dramatically accelerated clearance of Msm-Δ*clpC1*, reducing bacterial burden to approximately 2.5 log_10_ CFU at day 12 and attenuating lung inflammation (Fig. 4I and J). Conversely, iron chelation with DFO significantly rescued Msm-Δ*clpC1* survival, increasing CFU counts by over 1.5 log_10_ units and exacerbating inflammatory damage. In contrast, wild-type bacterial loads and pathology remained largely unaffected by either iron elevation or depletion (Fig. 4J and K). These results confirm that the survival of ClpC1-deficient mycobacteria is exquisitely sensitive to host iron status, and that iron-mediated toxicity underlies its *in vivo* clearance.

### ClpC1 and ClpX defects enhance mycobacterial susceptibility to antimicrobial drugs

We further assessed the antibiotic sensitivity of the respective knockdown strains. MIC assays showed that ClpC1 depletion substantially increased sensitivity to multiple drugs, reducing MICs by 2- to 32-fold (Table 1). Notably, unlike in other bacteria where ClpX impairment induces resistance to cell wall-targeting antibiotics (39, 40), *clpX* knockdown in mycobacteria did not confer such resistance to cell wall inhibitors (e.g., INH and EMB); instead, it resulted in even greater hypersensitivity, suggesting that targeting Clps could significantly boost the efficacy of antimycobacterial drugs. The MIC of Ecu, a ClpC1-targeting compound, for both Msm-WT and Msm-Δ*clpX* strains was 6.25 μg/mL (41). However, the Msm-Δ*clpC1* strain, with reduced ClpC1 levels, exhibited four-fold Ecu resistance. Notably, although Msm-Δ*clpC1* was hypersensitive to DTT alone (MIC 0.25 μg/mL vs 2 μg/mL for Msm-WT and Msm-Δ*clpX*), DTT did not restore its Ecu sensitivity; conversely, DTT enhanced Ecu’s activity against WT and *clpX* knockdown strains.

**TABLE 1:** MIC of Msm-WT, Msm-Δ*clpC1*, and Msm-Δ*clpX* strains in 7H9-OADC medium

7H9-OADC	Msm-WT	Msm-Δ*clpC1*	Msm-Δ*clpX*
Inhibitor	MIC (μg/mL)	MIC (μg/mL)	MIC (μg/mL)
INH	50	6.25	3.125
EMB (ethambutol)	0.5	0.0625	<0.0625
RIF	3.125	1.56	0.78
Capreomycin	3.125	0.78	<0.39
Erythromycin	40	<1.25	<1.25
Ecumicin	6.25	25	6.25
DTT	2	0.25	2
Ecu+DTT	3.125	25	3.125

**Fig 5:**
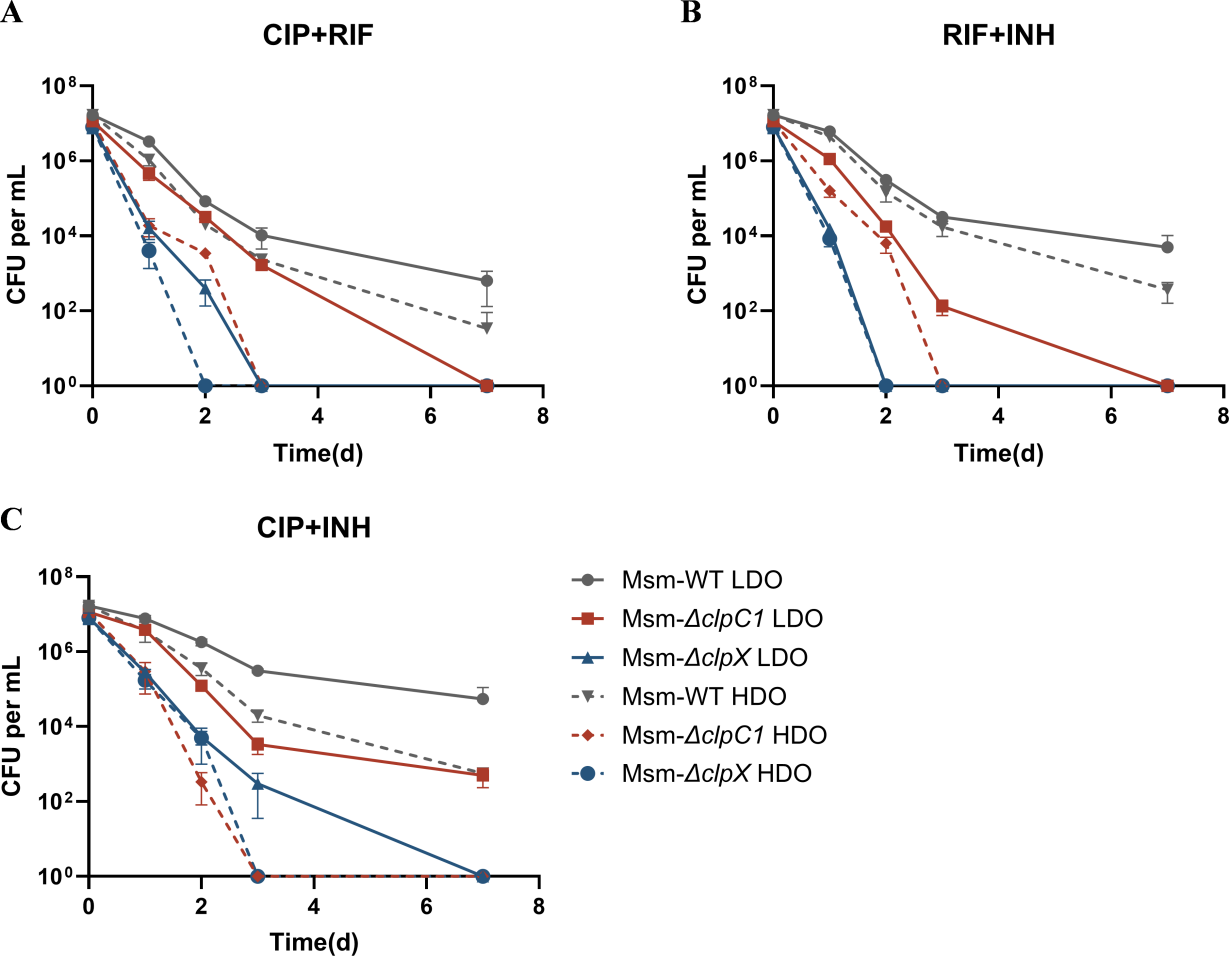
Combined drug bactericidal experiments with Msm-Δ*clpC1* and Msm-Δ*clpX* strains under different conditions. Bactericidal curves of Msm-WT, Msm-Δ*clpC1*, and Msm-Δ*clpX* strains cultured under different dissolved oxygen conditions and treated with combined antibiotics: CIP and RIF (**A**), RIF and INH (**B**), and CIP and INH (**C**) in 7H9-OADC medium. CIP, ciprofloxacin; RIF, rifampicin; INH, isoniazid; CFU, colony-forming units.

We evaluated the bactericidal effects of common antibiotic combinations under HDO and LDO. Consistent with reports that enhanced respiration promotes antibiotic efficacy (42), HDO significantly increased killing by all tested drug combinations (Fig. 5A through C). This phenomenon was most pronounced in Msm-Δ*clpC1*: the CIP and RIF combination achieved complete sterilization (<10^1^ CFU/mL) by day 3 under HDO, far exceeding LDO clearance rates (Fig. 5A). Similarly, with RIF + INH (Fig. 5B), Msm-Δ*clpC1* was eradicated within 3 days under HDO versus residual bacteria (~133 CFU/mL) under LDO (Fig. 5B). In the CIP + INH group (Fig. 5C), HDO enabled eradication by day 3, while ~500 CFU/mL remained on day 7 under LDO (Fig. 5C). Due to the severe growth defect, Msm-Δ*clpX* was eliminated by day 2 (<10^1^ CFU/mL) regardless of oxygen levels. These results indicate that targeting Clp proteins can significantly enhance antibiotic efficacy against mycobacteria, and conditions favoring the Fenton reaction, like increased dissolved oxygen, can further potentiate killing, particularly targeting ClpC1.

### Comparative proteomic analysis reveals distinct ClpC1- and ClpX-regulated networks in mycobacteria

**Fig 6:**
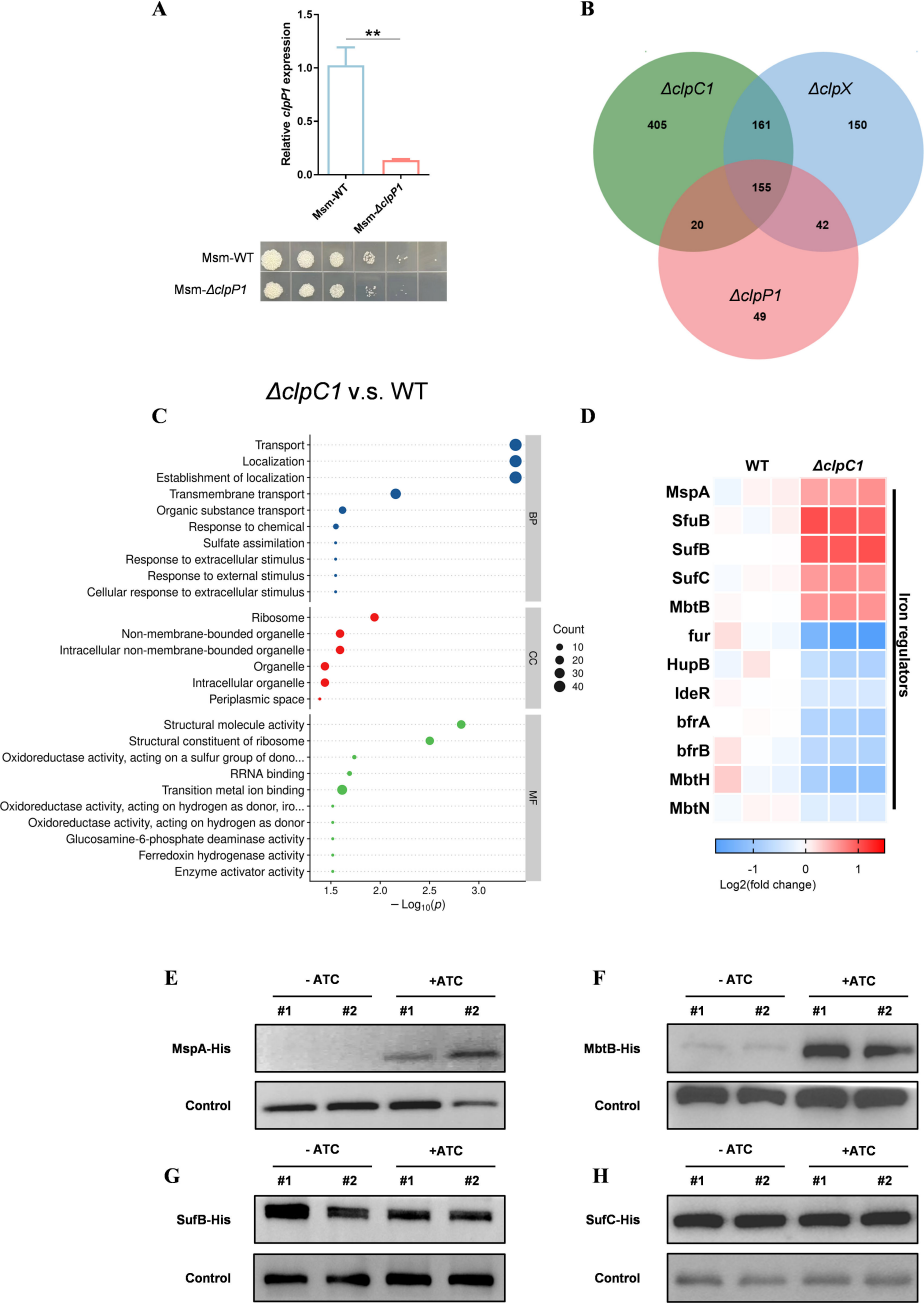
Proteomic analysis reveals selective regulation of mycobacterial functions by different Clp subunit combinations. (**A**) Validation of *clpP1* mRNA levels in Msm-Δ*clpP1*, accompanied by a spot assay illustrating growth differences compared to Msm-WT (**P < 0.01). (**B**) Venn diagram showing proteome overlaps among Msm-Δ*clpC1* (green), Msm-Δ*clpX* (blue), and Msm-Δ*clpP1* (pink), as determined by TMT-based proteomics (43). (**C**) Comparative GO enrichment analysis of differentially expressed proteins in Msm-Δ*clpC1* versus WT. (**D**) Heatmaps displaying differential protein expression (log₂ fold change) in Msm-Δ*clpC1* relative to WT. (**E–H**) Western blot analysis displaying the specific accumulation of MspA-His (**E**) and MbtB-His (**F**) proteins in Msm-Δ*clpC1* strains upon ATC induction, whereas SufB-His (**G**) and SufC-His (**H**) levels remained unchanged. Validated in two independent biological replicates (#1 and #2).

To investigate whether the functions of ClpC1 and ClpX are fully dependent on the Clp system, we constructed a conditional *clpP1* knockdown strain (Msm-Δ*clpP1*) (Fig. 6A) and performed TMT-labeled quantitative proteomics to compare the global profiles of Msm-Δ*clpC1*, Msm-Δ*clpX*, and Msm-Δ*clpP1* (Fig. 6B) (Table S1). Analysis revealed 155 coregulated proteins (61 upregulated and 92 downregulated) across all three mutants, with highly consistent regulatory trends, highlighting the broad responsive role of the Clp complex in maintaining proteostasis. In addition, 451 proteins were uniquely or commonly upregulated in the knockdown strains. Given that the Clp system primarily mediates ATP-dependent proteolysis, the upregulated proteins are presumed to be its potential degradation substrates. Functional annotation revealed that these upregulated proteins are primarily involved in key processes like cell wall biosynthesis, energy metabolism, transport, redox homeostasis, and amino acid metabolism, highlighting the physiological importance of the Clp system and diverse substrate recognition strategies. We confirmed the upregulation of known substrates Hsp20 and CarD in the Msm-Δ*clpC1* and Msm-Δ*clpP1* strains, respectively (23, 24). Previous studies indicate that ClpC1 can initiate substrate degradation by recognizing disordered sequences within the N- or C-termini of target proteins (24). Supporting this recognition mechanism, about 79.9% of upregulated proteins in the Msm-Δ*clpC1* group contained a disorder-promoting residue proportion of ≥50% within the N/C-terminal 15 amino acids.

To further clarify the functional differences between ClpC1 and ClpX, we performed comparative proteomic analyses of their respective knockdown strains versus WT. In Msm-Δ*clpX*, 508 proteins were differentially expressed, with 253 upregulated and 255 downregulated (Table S2). GO analysis revealed enrichment in cellular component-related terms, linking ClpX to cell structure and complex organization (Fig. S6A). Notably, key lipid transporters (MmpL4/5, MmpS1) (44), fatty acid metabolic enzymes (FadJ, FadE5/19/36, FadD28) (45–50), and MCE family proteins (MSMEG_0352, MSMEG_0353, MSMEG_5207), associated with lipid transport (51), were upregulated, while the repressor FadR_8 was downregulated, indicating disrupted lipid metabolism and cell envelope integrity. Furthermore, all detected universal stress proteins (MSMEG_3950, MSMEI_3854, MSMEG_5245, and MSMEG_3940) (52) were downregulated (Fig. S6B), suggesting impaired stress adaptation. These results demonstrate that ClpX depletion primarily perturbs cell wall lipid homeostasis and global protein stability.

In contrast, ClpC1 depletion altered 741 proteins, with 367 upregulated and 374 downregulated (Table S3), predominantly enriched in biological processes such as metal ion transport and stress response (Fig. 6C). Crucial iron homeostasis regulators displayed pronounced changes: key factors for Fe-S assembly (SufB, SufC), iron ion acquisition (the porin MspA [53]) and MbtB, a mycobactin synthase component (54), were markedly upregulated, while key iron uptake repressors (Fur, IdeR) (55, 56) and iron storage proteins (BfrA/B, HupB) (54) were strongly downregulated (Fig. 6D). This dysregulation drives the elevation of intracellular free Fe²^+^ (Fig. 4B), confirming that ClpC1’s central role in coordinating iron metabolism and metal ion homeostasis.

Based on ClpC1’s proteolytic function, we hypothesized that the upregulation of specific factors might result from impaired degradation. To identify genuine substrates among the candidates, we examined the post-translational stability of His-tagged MspA, MbtB, SufB, and SufC in the ClpC1-depleted background. Western blot analysis revealed that ClpC1 depletion specifically led to the accumulation of MspA and MbtB (Fig. 6E and F), confirming them as direct substrates. In contrast, SufB and SufC levels remained unchanged (Fig. 6G and H), indicating their elevation is likely an indirect response.

Additionally, the depletion of both ClpX and ClpC1 led to similar disturbances in a large number of dormancy-associated proteins (17, 57, 58) (e.g., DosR, HspX, Tgs1, Table S4), suggesting potential roles for ClpC1 and ClpX in mycobacterial dormancy regulation. However, only ClpC1 depletion specifically altered key reactivation markers, upregulating resuscitation-promoting factor RpfA (59) and downregulating sigma factor H (57). Msm-Δ*clpC1* is more sensitive to changes in oxygen concentration (Fig. 2C), and given that, TB relapse involves the transition of Mtb from hypoxic to oxygen-rich environments (60), we hypothesized that ClpC1 may be essential for resuscitation following hypoxia. To investigate this, we compared proteomic changes associated with ClpC1 deficiency under high and low dissolved oxygen conditions (CHDO/CLDO) and compared them with published Mtb proteomic profiles of oxygen-exposure resuscitation (61). Oxygen shifts induced dramatic proteomic alterations in the *clpC1* knockdown strain, affecting 1,036 proteins (596 upregulated, 440 downregulated) (Table S5). Key physiological processes such as energy metabolism (e.g., succinate dehydrogenase subunits SdhA/B/D) (62) and protein synthesis (e.g., ribosomal proteins RpsA/C, RpoB/C, and RplB) (63), were broadly repressed. Importantly, ClpC itself was specifically upregulated under oxygen exposure, unlike ClpX (Table S6). This finding is consistent with published proteomic data of Mtb resuscitation (64, 65), confirming the unique role of ClpC1 in oxygen sensing and reactivation.

## DISCUSSION

In this study, by systematically comparing *clpC1* and *clpX* conditional knockdowns, we reveal a clear functional division within the mycobacterial Clp system. While both unfoldases are essential for bacterial viability, ClpC1 and ClpX deficiencies provoke distinct yet partially overlapping alterations of central metabolic and stress response networks.

Phenotypically, in contrast to severe growth arrest observed in the *clpX* knockdown strain, the defect of the ClpC1-deficiency strain was uniquely dependent on environmental oxygen levels and closely associated with elevated intracellular Fe^2+^ (Fig. 4B). Our quantitative proteomic analysis traced this iron dysregulation to the synergistic accumulation of two direct ClpC1 substrates: the outer membrane iron porin MspA (53) and the siderophore biosynthetic enzyme MbtB (54). This dual accumulation is likely to drive iron overload by simultaneously enhancing extracellular iron uptake (via MspA) and siderophore-mediated iron scavenging (via MbtB). Consequently, the resulting iron surplus may induce a compensatory cellular response, as reflected in the upregulation of Fe-S cluster biosynthesis components (SufB, SufC) and the downregulation of iron storage proteins (BfrA/B, HupB). This shift from storage to utilization is further amplified by the elevated levels of iron reduction enzymes, such as ferredoxin-NADP^+^ reductase and pyruvate-ferredoxin oxidoreductase, which promote the reduction of Fe³^+^ to the more reactive Fe²^+^ (66, 67). Although Δ*clpX* also showed upregulation of SufB/SufC and downregulation of BfrB, it did not exhibit significant free Fe²^+^ accumulation (Fig. 4B), which may be due to the simultaneous upregulation of MmpL/MmpS family transporters in these cells. Previous studies have shown that MmpS4 and MmpS5, along with their corresponding MmpL4 and MmpL5 transporters, form a novel mycobactin export system, facilitating transport of mycobactin and carboxymycobactins (44). Notably, while TMT-based proteomics reveals global changes following Clp impairment, it cannot differentiate direct substrates from proteins indirectly affected through regulatory networks or compensatory mechanisms, including those that arise in response to long-term Clp depletion. Thus, both up- and down-regulated proteins may arise from secondary effects rather than direct Clp proteolysis. Biochemical validation, as performed here for MspA and MbtB, is therefore crucial to confirm direct Clp substrates. It is important to note that, in our assays, all recombinant proteins were fused with C-terminal His tags, which could potentially mask C-terminal degrons or recognition motifs, for example, in SufB and SufC.

Disruption of ClpX led to profound dysregulation of lipid metabolism (Fig. 6D). In the *clpX* knockdown strain, we observed marked upregulation of β-oxidation enzymes and downregulation of the fatty acid metabolism repressor FadR, along with a collective upregulation of MCE-family lipid transporters, including both the Mce1 and Mce4 complexes. Mce1 forms a functional ABC transporter complex, creating a hydrophobic channel that spans the mycobacterial cell envelope to directly facilitate lipid translocation (51). The Mce 4 transporter is a key system for cholesterol and lipid import across the cell wall (68). These alterations in the *clpX* knockdown strain are indicative of lost homeostatic control over fatty acid catabolism and cell envelope lipid trafficking. This may ultimately destabilize the balance between lipid storage and consumption—a balance critical to the survival of mycobacteria, even dormant bacteria, as Mtb dormancy critically depends on alternative carbon and energy sources such as host-derived cholesterol and fatty acids (69, 70).

Importantly, ClpC1 or ClpX deficiency resulted in mutual downregulation of dormancy-related regulators, including DosR, HspX, and Tgs1. Further, we observed extensive alterations in energy metabolism in both ClpC1- and ClpX-deficient strains, as key components of energy and central carbon metabolism, such as CydC/D, P-type ATPases, and isocitrate lyase (ICL), were suppressed (71–73). Consistently, we observed reduced ATP levels detected in the ClpC1 and ClpX impaired strains (Fig. S7A). Notably, CydDC expression is usually upregulated in response to hypoxia or NO and is critical during chronic infection *in vivo,* while ICL catalyzes the first step of the glyoxylate shunt, which is pivotal for assimilation of fatty acids and persistence of Mtb. These findings indicate that both ClpC1 and ClpX deficiencies may lead to an energy-deficient state incompatible with long-term survival.

Additionally, both *clpC1* and *clpX* knockdowns exhibited pronounced oxidative stress and increased susceptibility to ROS-mediated damage. These disruptions were accompanied by significant decreases in EGT concentration (Fig. S4A), downregulation of the trehalose synthesis pathway (such as treY and treZ), and a marked reduction in trehalose levels (Fig. S7B), which are two key redox and energy-buffering systems (33, 73). Maintenance of redox homeostasis is a cornerstone of Mtb dormancy, further underscoring the essential role of ClpC1 and ClpX in orchestrating metabolic adaptations required for the dormant state. However, we tend to believe that ClpC1 plays a role in bacterial reactivation, as ClpC1 (rather than ClpX) exhibited specific upregulation under oxygen exposure, and the resuscitation key markers were upregulated in the ClpC1 deficient strain (Table S4). Although protein synthesis and energy production were partially initiated upon oxygen exposure in the ClpC1-deficient strain, which was evidenced by upregulation of several core biosynthetic and energy-producing proteins (AtpD/MSMEG_4936, AtpC/MSMEG_4935), the translation initiation factor InfB, and the TCA cycle enzyme Icd2 (74–77), iron dysregulation, Fenton-driven oxidative damage, and downregulation of key effectors required for oxidative damage repair ultimately prevented Mtb’s successful resuscitation.

**Fig 7:**
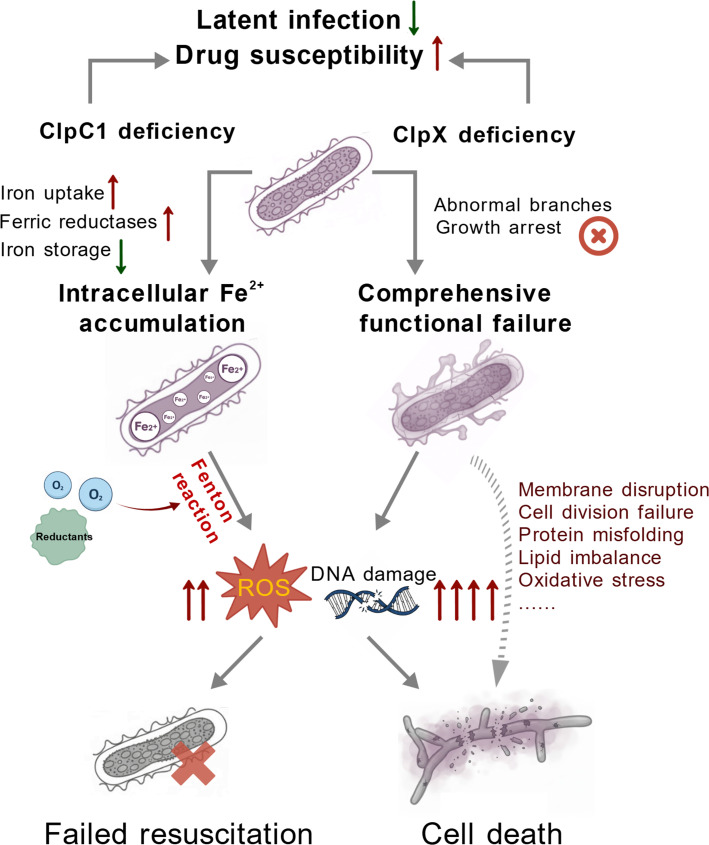
Schematic representation of divergent molecular pathologies induced by ClpC1 and ClpX deficiencies. ClpC1 deficiency (left) triggers the specific accumulation of substrates MspA and MbtB, which drives dysregulated iron uptake and intracellular iron overload. This iron imbalance fuels the Fenton reaction, generating a ROS burst that results in two distinct outcomes: attenuated survival (both *in vitro* and *in vivo*) and DNA damage-induced failure of resuscitation. ClpX deficiency (right) causes comprehensive functional failure, manifesting as abnormal morphology and growth arrest, along with constitutive oxidative stress and severe DNA damage, ultimately leading to cell death. Both ClpC1- and ClpX-deficient strains exhibit increased bacterial susceptibility to drugs and may impact Mtb latent infection. The pattern was created with BioGDP.com (78).

In summary, our study systematically dissects the distinct and essential roles of the Clp unfoldases ClpX and ClpC1 in mycobacteria. We establish ClpX as a guardian of core cellular integrity, while unveiling a pivotal function for ClpC1 in orchestrating iron homeostasis and bacterial adaptation to fluctuating oxygen—a key determinant of resuscitation during infection (Fig. 7). It is important to acknowledge the limitations of our experimental models. Due to BSL-3 requirements, we used non-pathogenic Msm and attenuated Mtb H37Ra instead of the fully virulent H37Rv strain. While the use of Msm in animal studies allowed us to examine the iron sensitivity of ClpC1-deficient mycobacteria, these models do not fully capture the pathogenicity and host interactions of virulent Mtb. Further validation in virulent strains and advanced animal models will therefore be necessary.

By demonstrating that ClpC1 deficiency selectively impairs bacterial survival under iron stress, we identify iron acquisition as a potential therapeutic vulnerability in mycobacteria. Siderophore-antibiotic conjugates such as cefiderocol exemplify how iron uptake pathways can be leveraged for antibacterial strategies (79). Thus, targeting ClpC1-mediated iron regulation may inform the development of new approaches for tackling persistent mycobacterial infection and may also be relevant in other pathogens like *Clostridioides difficile*, where ClpC is also essential for virulence (80). Overall, our findings highlight the Clp system as a potential therapeutic target whose inhibition could affect different aspects of mycobacterial survival. These insights may help guide future research into more effective ways to target both active and persistent mycobacteria.

## Data Availability

The mass spectrometry proteomics data generated in this study have been deposited to the ProteomeXchange Consortium via the iProX partner repository with the data set identifier PXD071293. Source data for Fig. 1 to 7 are provided with this paper (or in the supplemental material). All other data supporting the findings of this study are available within the article and its supplemental material or from the corresponding author upon reasonable request.

## SUPPLEMENTAL MATERIAL

Supplemental figures spectrum.00496-26-s0001.pdf Fig. S1 to S6.

Supplemental material spectrum.00496-26-s0002.docx Strains, plasmids, and primers.

Table S1 spectrum.00496-26-s0003.xlsx Proteins co-downregulated across clpC1, clpP, and clpX knockdown strains.

Table S2 spectrum.00496-26-s0004.xlsx Proteins up/downregulated in the clpX knockdown strain compared to WT.

Table S3 spectrum.00496-26-s0005.xlsx Proteins up/downregulated in the clpC1 knockdown strain compared to WT.

Table S4 spectrum.00496-26-s0006.xlsx Dormancy protein dysregulation in clpC1 and clpX knockdown strains.

Table S5 spectrum.00496-26-s0007.xlsx Proteins up/downregulated in the clpC1 knockdown strain under HDO versus LDO.

Table S6 spectrum.00496-26-s0008.xlsx Proteins exhibiting different and concordant expression trends in the ClpC1-HDO/LDO comparison and the Resuscitation Group.

ASM does not own the copyrights to Supplemental Material that may be linked to, or accessed through, an article. The authors have granted ASM a non-exclusive, world-wide license to publish the Supplemental Material files. Please contact the corresponding author directly for reuse.
