# Supplementary material for: ClpC1-dependent iron homeostasis underlies mycobacterial defense against oxygen-driven Fenton reaction during reactivation
Source: Microbiol Spectr. 2026 Jun 18;14(8):e00496-26. doi: 10.1128/spectrum.00496-26 (PMC13435763; doi:10.1128/spectrum.00496-26)
Supplement: Supplementary file 1 [file spectrum.00496-26-s0001.pdf]

ClpC1-Dependent Iron Homeostasis Underlies Mycobacterial Defense Against Oxygen-Driven Fenton Reaction During Reactivation

Mingzhe Chi[1], Yuqing Zheng[1], Wenkai Zheng[1], Youran Chen[1], Bohong Zhou[1], Jiayu Chen[1], Jiacheng Bai[1], Yuchang Di[1,4,5], Xuelian Zhang[1,2,3*]

[1] State Key Laboratory of Genetics and Development of Complex Phenotypes, School of Life Sciences, Institute of Infection and Health, Fudan University, No. 2005 Songhu Road, Shanghai, 200433, China.

[2] Shanghai Sci-Tech Inno Center for Infection & Immunity, No. 1688 Guoquan Bei Road, Shanghai 200438, China

[3] MOE Engineering Research Center of Gene Technology, Shanghai Engineering Research Center of Industrial Microorganism, School of Life Sciences, Fudan University, Shanghai 200433, China

[4] Infectious Disease Department, Shenzhen Children's Hospital, Shenzhen, People's Republic of China

[5] Department of Infectious Diseases, Shanghai Key Laboratory of Infectious Diseases and Biosafety Emergency Response, National Medical Center for Infectious Diseases, Huashan Hospital, Fudan University, Shanghai, China

CONTACT Xuelian Zhang, xuelianzhang@fudan.edu.cn

Supplementary information consists of 7 figures (Figure S1-S7), 6 tables (Table S1-S6) and Supplementary Figure Legend

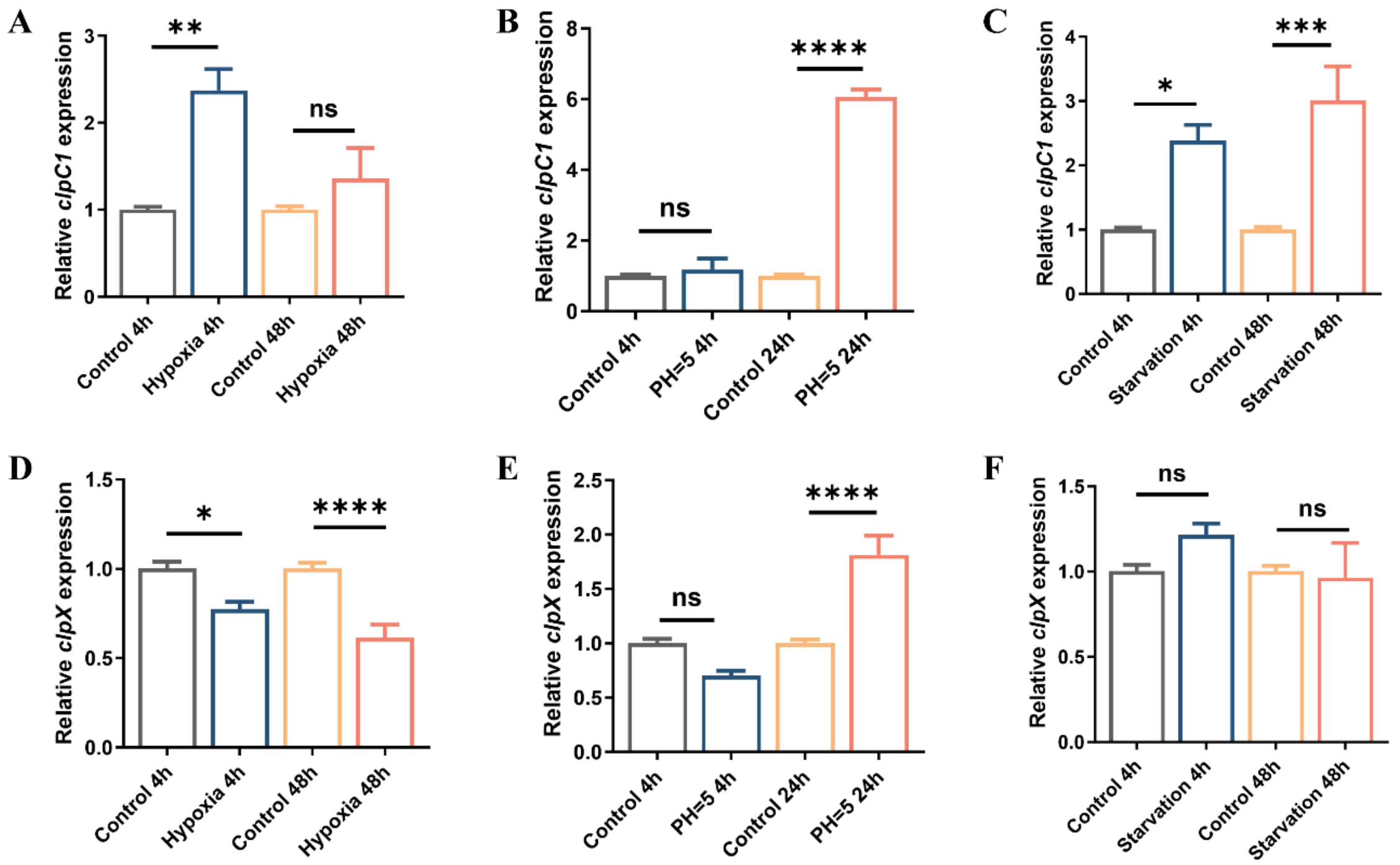

Fig S1. Differential expression of *clpC1 and clpX* under different stresses. (A–F) RT-qPCR analysis of *clpC1* and *clpX* mRNA levels in M.tb to hypoxia (A, D), low pH (B, E), or starvation (C, F) for indicated time points. All samples were cultured in 7H9-OADC medium, and gene expression was normalized to the reference gene sigA. Relative expression is shown compared to the respective time-matched control group. Data are presented as mean ± SD of three biological replicates. Statistical significance was determined by one-way ANOVA with Tukey's post-hoc test (*$p < 0.05$; **$p < 0.01$; ***$p < 0.001$; ****$p < 0.0001$; ns: not significant).

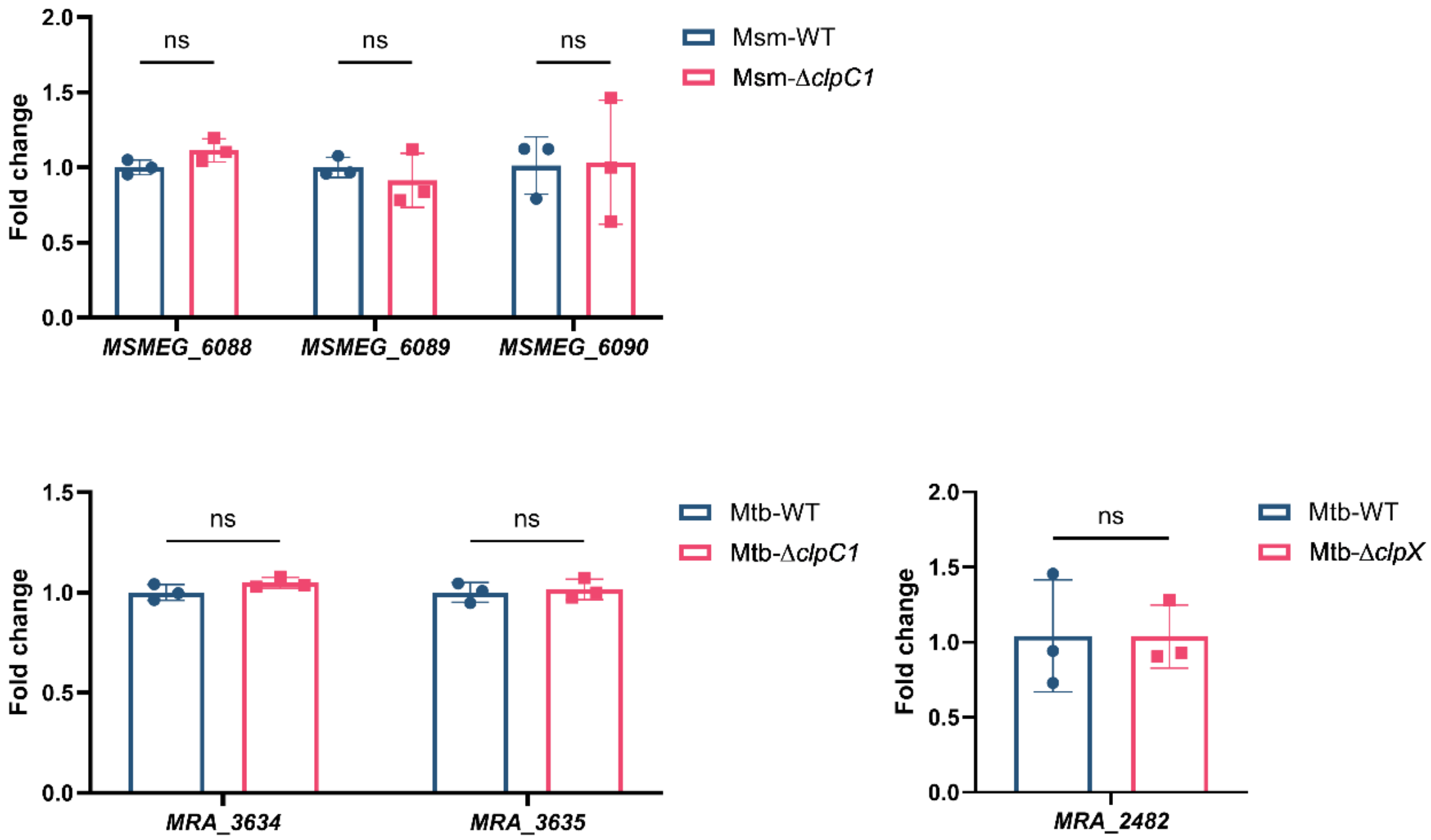

Figure S2. Evaluation of potential polar effects. Evaluation of potential polar effects. RT-qPCR analysis of genes downstream of targeted *clp* genes in Msm (*clpC1*: *MSMEG_6088, MSMEG_6089, MSMEG_6090*) and Mtb (*clpC1*: *MRA_3634, MRA_3635*; *clpX*: *MRA_2482*). No significant differences were observed between WT and knockdown strains, confirming the absence of CRISPRi polar effects (ns, not significant).

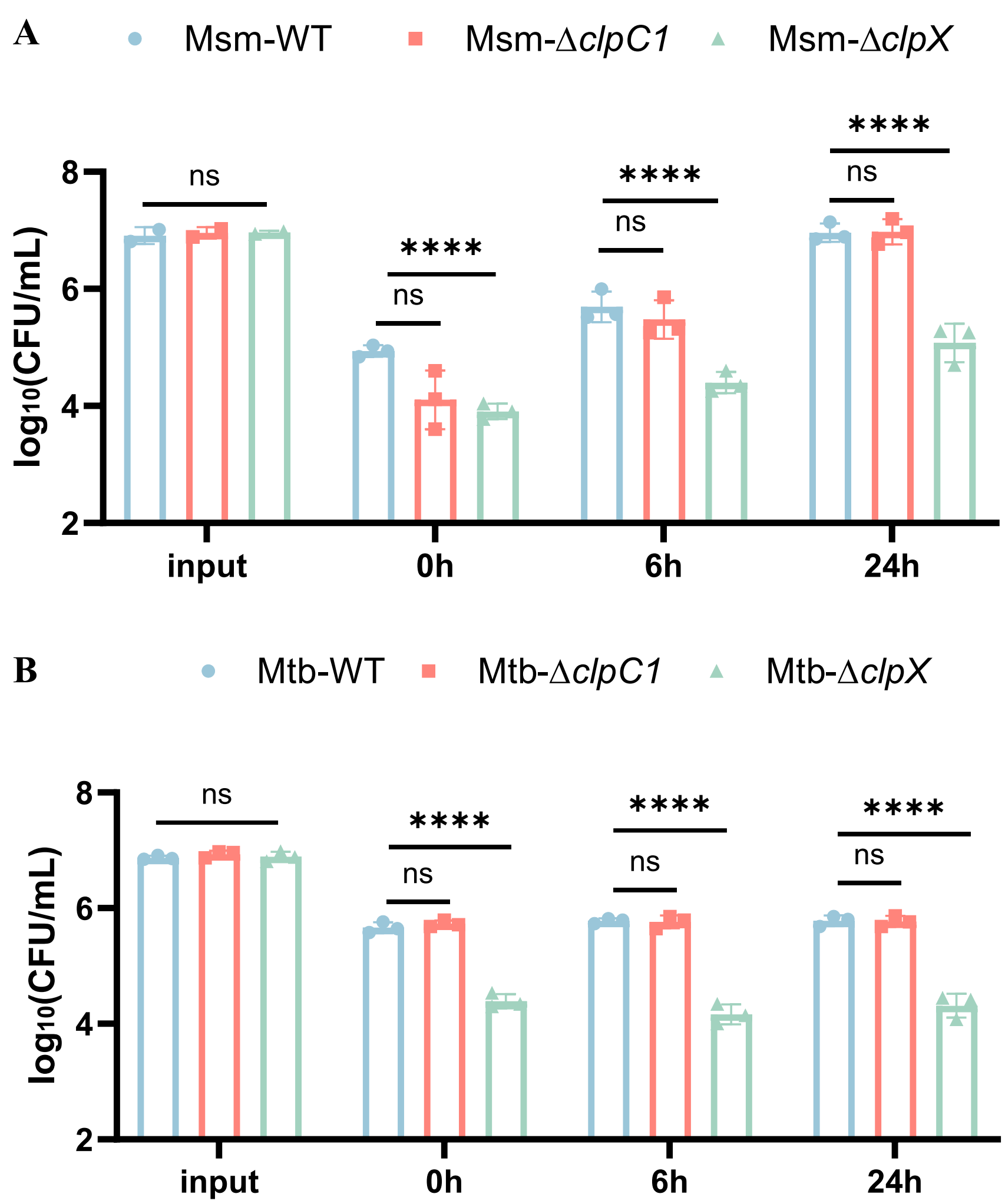

Fig S3. Intracellular survival of ClpC1 and ClpX knockdown strains in THP-1 cells. (A, B) CFU of Msm (A) and Mtb (B) WT, *ΔclpC1*, and *ΔclpX* strains following infection of differentiated THP-1 cells. Bacterial counts were measured at the indicated time points post-infection. Data are presented as $\log_{10}$(CFU/mL) and represent mean ± SD from three biological replicates. Statistical analysis was performed by one-way ANOVA with Tukey's post-hoc test (*$p < 0.05$; **$p < 0.01$; ***$p < 0.001$; ****$p < 0.0001$; ns, not significant).

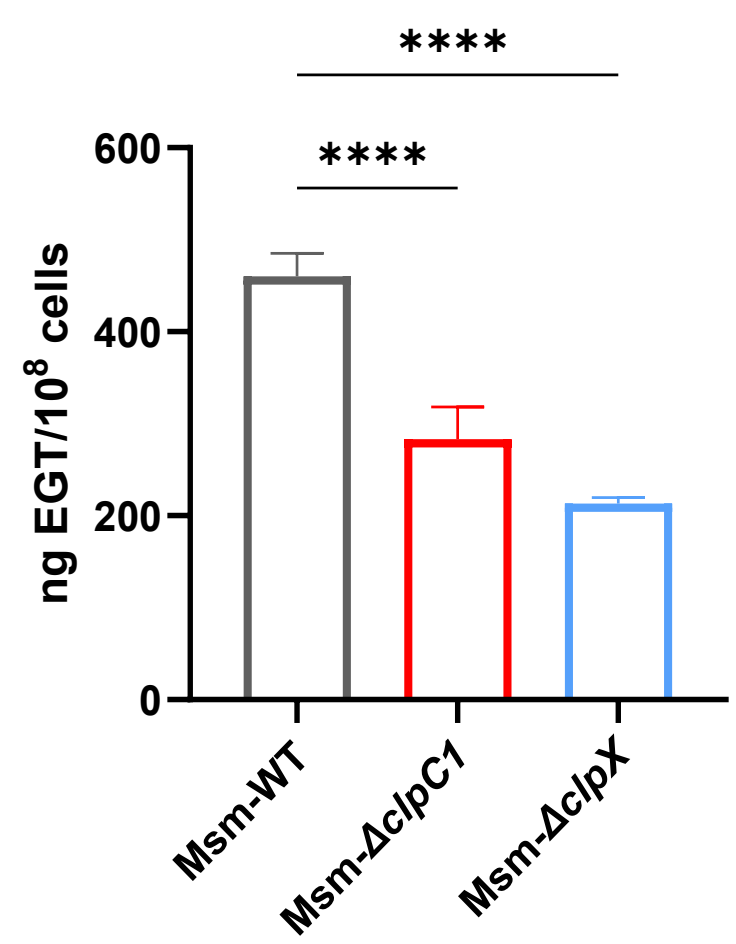

Figure S4. Intracellular EGT content in Msm-WT, Msm-*ΔclpC1*, and Msm-*ΔclpX* strains, measured by LC-MS. Statistical significance was evaluated by one-way ANOVA followed by Tukey's multiple comparison test (**p < 0.01; ***p < 0.001; ****p < 0.0001).

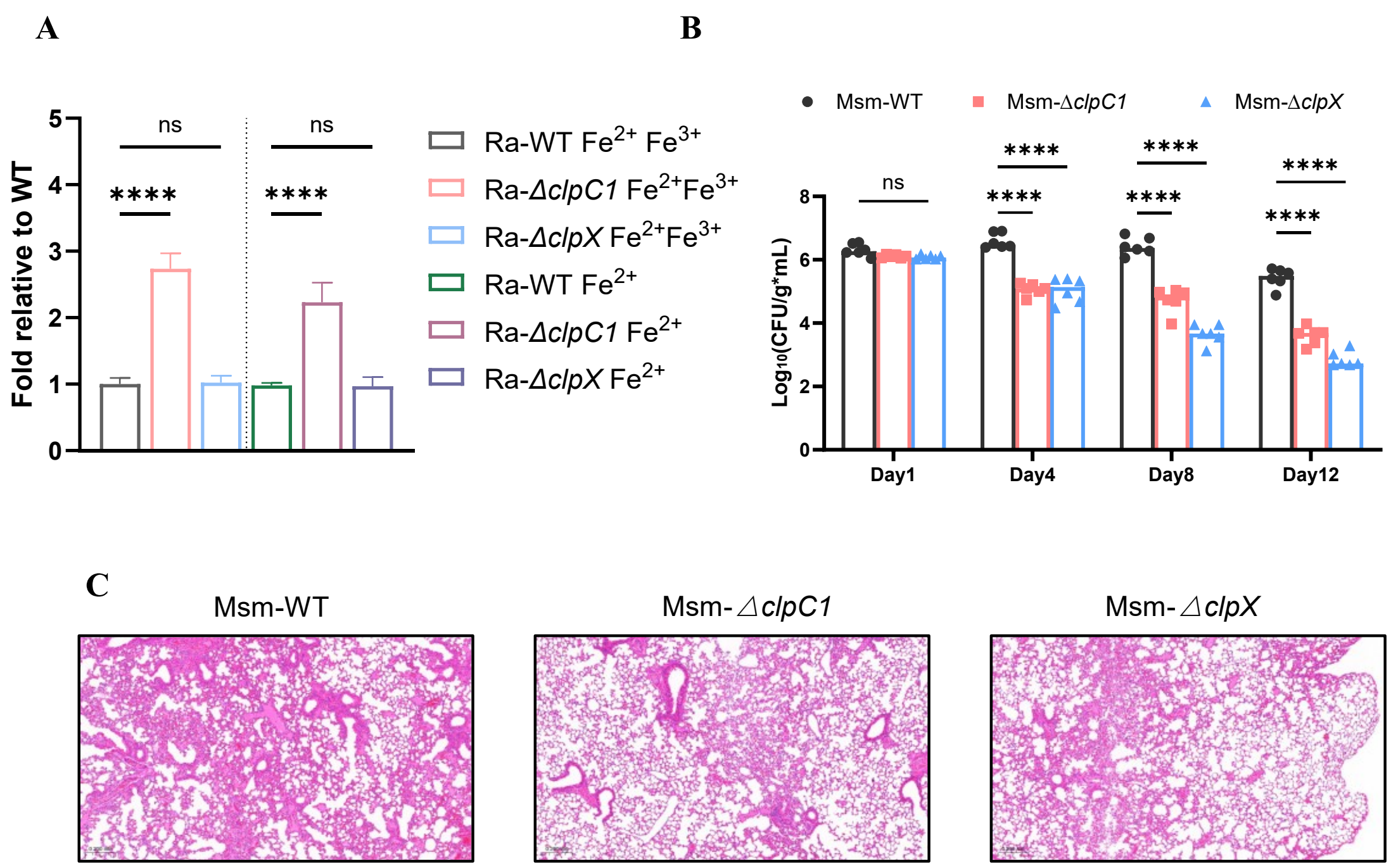

Figure S5. (A) Levels of bivalent (Fe^{2+}) and trivalent (Fe^{3+}) iron ions in Ra-WT, Ra-*ΔclpC1*, and Ra -*ΔclpX* strains in 7H9-OADC medium. (A) Survival of Msm-WT, Msm-*ΔclpC1*, and Msm-*ΔclpX* strains in C57BL/6 mice. Lung bacterial burdens were enumerated at days 1, 4, 8, and 12 post-infection. (B) H&E staining images of lung tissues collected at day 8 post-infection from mice infected with Msm-WT, Msm-*ΔclpC1*, or Msm-*ΔclpX* strains. (****$p < 0.0001$; ns, not significant).

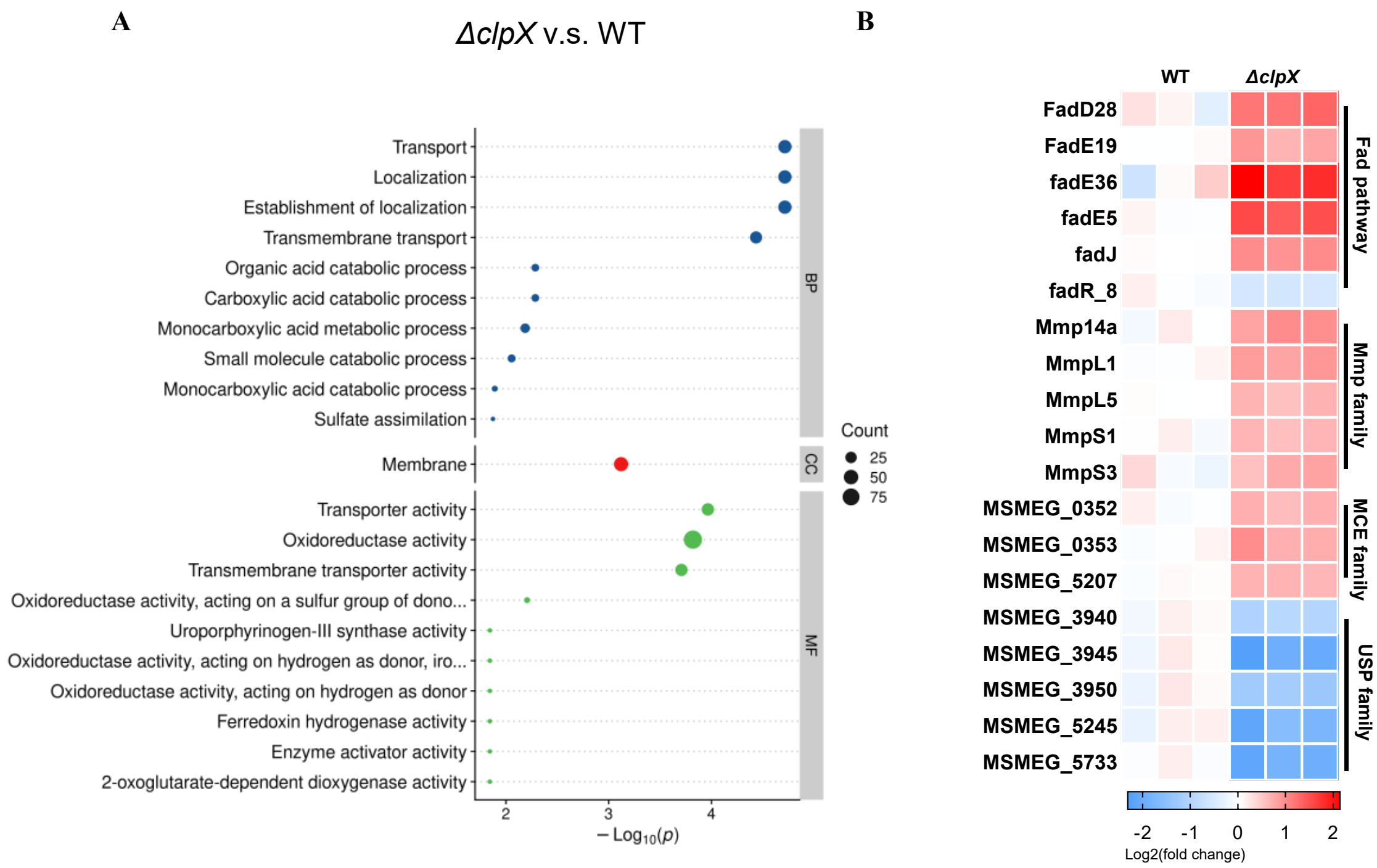

Fig S6. (A) Comparative GO enrichment analysis of differentially expressed proteins in Msm-*ΔclpX* versus WT. (B) Heatmaps displaying differential protein expression ($\log_2$ fold change) in Msm-*ΔclpX* relative to WT.

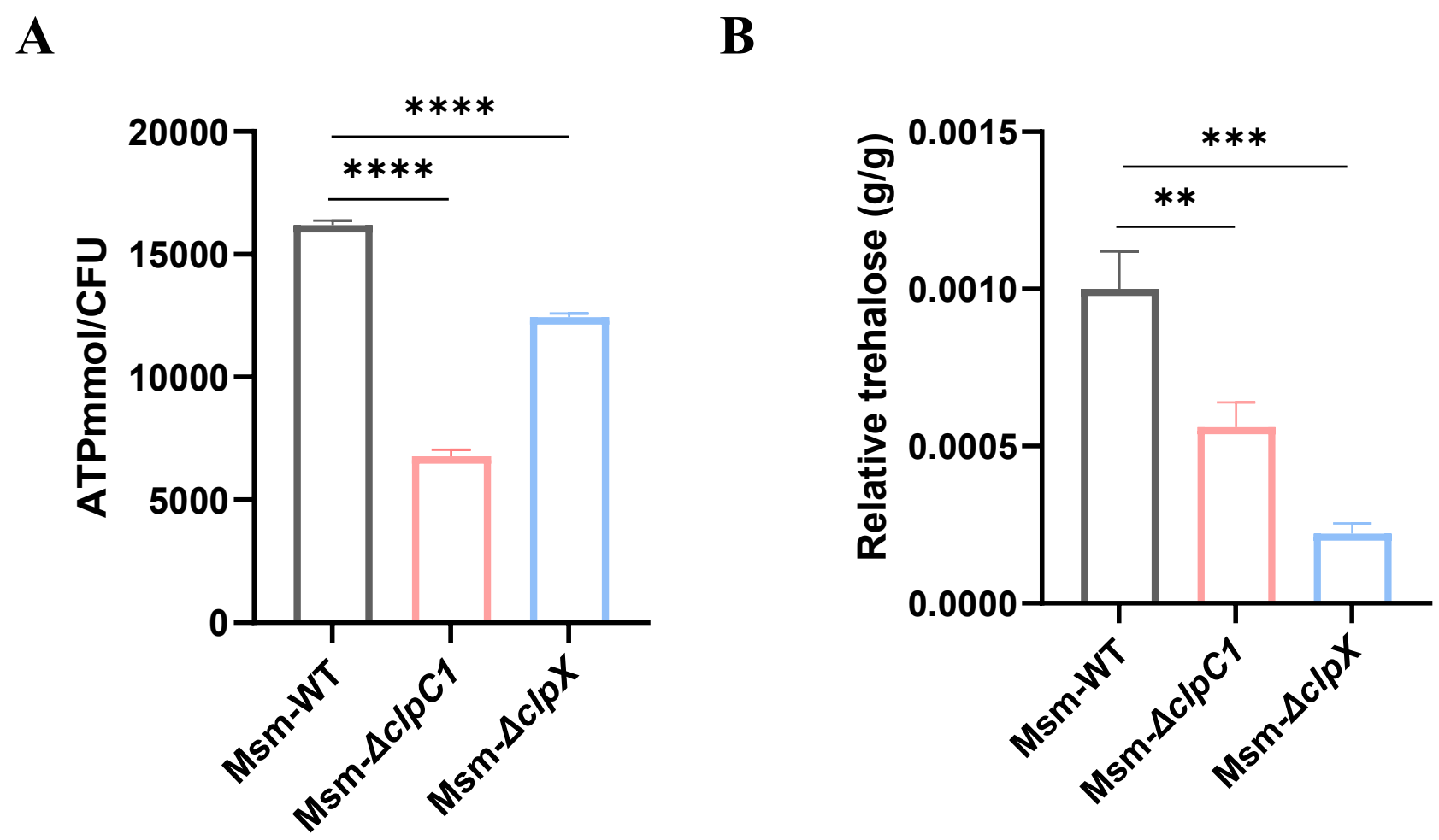

Figure S7. Disrupted redox and energy metabolism in *clpC1* and *clpX* knockdown strains (A) Intracellular ATP levels in the three strains. (B) Relative trehalose content in the three strains, normalized to bacterial cell mass (g/g), normalized to CFU. Data are presented as mean ± SD from three biological replicates. Statistical significance was evaluated by one-way ANOVA followed by Tukey's multiple comparison test (**$p < 0.01$; ***$p < 0.001$; ****$p < 0.0001$).
