# Supplementary material for: ClpC1-dependent iron homeostasis underlies mycobacterial defense against oxygen-driven Fenton reaction during reactivation
Source: Microbiol Spectr. 2026 Jun 18;14(8):e00496-26. doi: 10.1128/spectrum.00496-26 (PMC13435763; doi:10.1128/spectrum.00496-26)
Supplement: Supplementary file 2 [file spectrum.00496-26-s0002.docx]

**Strains, plasmids, and primers used in this study**

**1. Bacterial Strains**
*Escherichia coli DH5α* (Description: General cloning host) (Source: Sangon Biotech)

*Mycolicibacterium smegmatis* MC² 155 (Source: Laboratory stock)

*Mycobacterium tuberculosis* H37Ra (Source: Laboratory stock)

**2. Plasmids**

PLJR962 (Description: CRISPRi episomal vector, KanR, ATc-inducible) (Source: Laboratory stock)

PLJR965 (Description: CRISPRi integration vector, KanR, ATc-inducible) (Source: Laboratory stock)

PMV261 (Description: Mycobacterial overexpression vector, KanR) (Source: Laboratory stock) PMV261-hyg (Description: Modified PMV261, KanR replaced with HygR) (Source: This study)

**3.Primers**

**[sgRNA Oligos for CRISPRi Msm]**

clpC1-sgRNA-F (Sequence: GGGAGAGCGCGTCGTCGTCGCCGAA) (Description: Forward oligo for ClpC1 sgRNA)

clpC1-sgRNA-R (Sequence: AAACTTCGGCGACGACGACGCGCTC) (Description: Reverse oligo for ClpC1 sgRNA)

clpX-sgRNA-F (Sequence: GGGAGACCTGCTTTTGGCTCTTCCCA) (Description: Forward oligo for clpX sgRNA)

clpX-sgRNA-R (Sequence: AAACTGGGAAGAGCCAAAAGCAGGTC) (Description: Reverse oligo for clpX sgRNA)

clpP1-sgRNA-F (Sequence: TGCTGGCCGCCGAAGACGCC) (Description: Forward oligo for clpP1 sgRNA)

clpP1-sgRNA-R (Sequence: GGCGTCTTCGGCGGCCAGCA) (Description: Reverse oligo for clpP1 sgRNA)

**[sgRNA Oligos for CRISPRi Mtb]**

clpC1-sgRNA-F (Sequence: GGGACTCACCCACCTGCACCGGCT) (Description: Forward oligo for ClpC1 sgRNA)

clpC1-sgRNA-R (Sequence: AAACAGCCGGTGCAGGTGGGTGAG) (Description: Reverse oligo for ClpC1 sgRNA)

clpX-sgRNA-F (Sequence: GGGATGACCTGTTTTTGGCTCTTCCCGC) (Description: Forward oligo for clpX sgRNA)

clpX-sgRNA-R (Sequence: AAACGCGGGAAGAGCCAAAAACAGGTCA) (Description: Reverse oligo for clpX sgRNA)

**[RT-qPCR Primers Mtb]**

sigA-qPCR-F (Sequence: CCGTCGAGGAGCTCGACGCTGAACC) (Description: Forward primer for internal control sigA)

sigA-qPCR-R (Sequence: CTCAGCTTCGAGGTCTTCGTGGTCT) (Description: Reverse primer for internal control sigA)

clpC1-qPCR-F (Sequence: GTACATCGAGAAGGACGCCG) (Description: Forward primer for ClpC1 expression)

clpC1-qPCR-R (Sequence: ACCGGTCGTTGATGTAGCGG) (Description: Reverse primer for clpC1 expression)

clpX-qPCR-F (Sequence: GAACATCTTGATGCTCGGACCCA) (Description: Forward primer for clpX expression)

clpX-qPCR-R (Sequence: GATCTTGTCGACCTCGTCGATGT) (Description: Reverse primer for clpX expression)
q-MRA_3635-F (Sequence: ATCAGCGCGGCTAACGCG) (Description: Forward primer for MRA_3635)

q-MRA_3635-R (Sequence: GCTGAACGTTGGCGGCCTC) (Description: Reverse primer for MRA_3635)

q-MRA_3634-F (Sequence: ACCGGTGGCACTGGCGGTAC) (Description: Forward primer for MRA_3634)

q-MRA_3634-R (Sequence: CGTCACCCCCAACGCCTGGT) (Description: Reverse primer for MRA_3634)

q-MRA_2482-F (Sequence: CTGCTGAATTTCTCGTTGGC) (Description: Forward primer for MRA_2482)

q-MRA_2482-R (Sequence: AGCACCGAGATGGAATACAA) (Description: Reverse primer for MRA_2482)

**[RT-qPCR Primers Msm]**

sigA-qPCR-F (Sequence: CGTCCGGCGACTTCGTGT) (Description: Forward primer for internal control sigA)

sigA-qPCR-R (Sequence: TGGCCAGCTCCACCTCTTCT) (Description: Reverse primer for internal control sigA)

clpC1-qPCR-F (Sequence: GAAGGCCCACCAGGAGATCT) (Description: Forward primer for ClpC1 expression)

clpC1-qPCR-R (Sequence: TCGAGATGTCGGACGTGCCGA) (Description: Reverse primer for clpC1 expression)

clpX-qPCR-F (Sequence: GGACTCCGCCAAGCGGAC) (Description: Forward primer for clpX expression)

clpX-qPCR-R (Sequence: TCTGCGCCAGGTAGGTCTTG) (Description: Reverse primer for clpX expression)

clpP1-qPCR-F (Sequence: TCGACTCGGTGTACGAGCGGCT) (Description: Forward primer for clpP1 expression)

clpP1-qPCR-R (Sequence: GCGAGCACCATGGTGTCGTA) (Description: Reverse primer for clpP1 expression)

MSMEG_6088-qPCR-F (Sequence: TCACTCGGTGACATCGATCCAG) (Description: Forward primer for MSMEG_6088 expression)

MSMEG_6088-qPCR-R (Sequence: TGTCGATGCGCCAGAAGGAT) (Description: Reverse primer for MSMEG_6088 expression)

MSMEG_6089-qPCR-F (Sequence: TGATCAACCGGGCCATCGACT) (Description: Forward primer for MSMEG_6089 expression)

MSMEG_6089-qPCR-R (Sequence: CAGAACAACACCGCGAGCAG) (Description: Reverse primer for MSMEG_6089 expression)

MSMEG_6090-qPCR-F (Sequence: TGACTTCTCCGGCTCACCAG) (Description: Forward primer for MSMEG_6090 expression)

MSMEG_6090-qPCR-R (Sequence: GCACGTAGGTGTTGTTGCCGA) (Description: Reverse primer for MSMEG_6090 expression)

**[Cloning Primers for Overexpression]**

hyg-F (Sequence: ACTAGTGTGACACAAGAATCCCTGT) (Description: Forward primer to amplify hyg resistance gene)

hyg-R (Sequence: GCTAGCTCAGGCGCCGGGGGCGGTGT) (Description: Reverse primer to amplify hyg resistance gene)

mspA-His-F (Sequence: TTTGGATCCatgaaggcaatcagt) (Description: Forward primer for mspA cloning)

mspA-His-R (Sequence:TTTAAGCTTTCAGTGGTGGTGGTGGTGGTGGTTCATGTTCCAGGGTT) (Description: Reverse primer for mspA cloning with His-tag)

mbtB-His-F (Sequence: TTTGGATCCATGGGCGACGTCGCT) (Description: Forward primer for mbtB cloning)

mbtB-His-R (Sequence: TTTAAGCTTTCAGTGGTGGTGGTGGTGGTGCGATGTCGCGGC) (Description: Reverse primer for mbtB cloning with His-tag)

sufB-His-F (Sequence: TTTGGATCCATGACGACCACCCCC) (Description: Forward primer for sufB cloning)

sufB-His-R (Sequence: TTTAAGCTTTCAGTGGTGGTGGTGGTGGTGGCCGACCGCACC) (Description: Reverse primer for sufB cloning with His-tag)

sufC-His-F (Sequence: TTTGGATCCATGAGCACCCTGGAA) (Description: Forward primer for sufC cloning)

sufC-His-R (Sequence: TTTAAGCTTTCAGTGGTGGTGGTGGTGGTGGGCCCCTGCGGC) (Description: Reverse primer for sufC cloning with His-tag)
